# Desynchronization boost by non-uniform coordinated reset stimulation in ensembles of pulse-coupled neurons

**DOI:** 10.3389/fncom.2013.00063

**Published:** 2013-05-17

**Authors:** Leonhard Lücken, Serhiy Yanchuk, Oleksandr V. Popovych, Peter A. Tass

**Affiliations:** ^1^Department of Mathematics and Natural Sciences II, Institute of Mathematics, Humboldt University of BerlinBerlin, Germany; ^2^Institute of Neuroscience and Medicine - Neuromodulation (INM-7), Research Center JülichJülich, Germany; ^3^Department of Neuromodulation, Cologne Medical SchoolCologne, Germany

**Keywords:** desynchronization, pulse coupled neurons, coordinated reset stimulation, phase response curve, stimulation timing, cluster states

## Abstract

Several brain diseases are characterized by abnormal neuronal synchronization. Desynchronization of abnormal neural synchrony is theoretically compelling because of the complex dynamical mechanisms involved. We here present a novel type of coordinated reset (CR) stimulation. CR means to deliver phase resetting stimuli at different neuronal sub-populations sequentially, i.e., at times equidistantly distributed in a stimulation cycle. This uniform timing pattern seems to be intuitive and actually applies to the neural network models used for the study of CR so far. CR resets the population to an unstable cluster state from where it passes through a desynchronized transient, eventually resynchronizing if left unperturbed. In contrast, we show that the optimal stimulation times are non-uniform. Using the model of weakly pulse-coupled neurons with phase response curves, we provide an approach that enables to determine optimal stimulation timing patterns that substantially maximize the desynchronized transient time following the application of CR stimulation. This approach includes an optimization search for clusters in a low-dimensional pulse coupled map. As a consequence, model-specific non-uniformly spaced cluster states cause considerably longer desynchronization transients. Intriguingly, such a desynchronization boost with non-uniform CR stimulation can already be achieved by only slight modifications of the uniform CR timing pattern. Our results suggest that the non-uniformness of the stimulation times can be a medically valuable parameter in the calibration procedure for CR stimulation, where the latter has successfully been used in clinical and pre-clinical studies for the treatment of Parkinson's disease and tinnitus.

## 1. Introduction

Pathological neuronal synchronization is a hallmark of several neurological disorders like Parkinson's disease (PD), essential tremor, tinnitus, or epilepsy (Lenz et al., 1994; Nini et al., 1995; Mormann et al., 2000; Weisz et al., 2005, 2007; Kane et al., 2009; Schnitzler et al., 2009; Roberts et al., 2010), whereas the neuronal firing is uncorrelated in the normal state (Nini et al., 1995; Wilson et al., 2004) such that the abnormal synchronization is associated with pathology and symptoms (Levy et al., 2000). The standard therapy for medically refractory PD patients is electrical deep brain stimulation (DBS), where a high-frequency (HF >100 Hz) electrical pulse train is administered to target brain areas via chronically implanted depth electrodes (Benabid et al., 1991). HF DBS is found to significantly alter the neuronal activity of the stimulated neurons: The neuronal firing can be suppressed in the vicinity of the stimulation electrode, whereas the neurons are overactivated in the output structures of the stimulated neuronal population such that the pathological firing pattern is changed, see (Deniau et al., 2010) for review.

The mechanism of HF DBS is not fully understood. A modeling study by Wilson et al. (2011) suggests that the HF periodic DBS may induce a chaotic desynchronization, while a desynchronizing impact of a periodic forcing on synchronized populations seems to be a rather general phenomenon (Popovych and Tass, 2011). Also, as shown computationally, the effect of HF DBS strongly depends on the target structures stimulated (Hauptmann and Tass, 2007): Delivering HF DBS (nearly) exclusively to excitatory target structures may cause a desynchronization, whereas a stronger involvement of inhibitory target structures typically causes a pronounced inhibition. In some patients, however, HF DBS may be ineffective or cause side effects (Limousin et al., 1999; Kumar et al., 2003; Volkmann, 2004; Freund, 2005; Rodriguez-Oroz et al., 2005). Accordingly, along the lines of a model-based approach (Tass, 1999) novel stimulation techniques have been developed (Tass, 2001a, 2003a,b; Rosenblum and Pikovsky, 2004; Hauptmann et al., 2005; Popovych et al., 2005, 2006; Pyragas et al., 2007; Popovych and Tass, 2010), which selectively counteract the pathological synchronization and restore uncorrelated neuronal firing. They are based on either phase resetting or feedback approaches, where from a theoretical standpoint the latter might have a great potential in controlling pathological synchronization, but so far have not been technically realized.

Other methods suggest to stimulate a single oscillator in the network (Nabi and Moehlis, 2011), drive the neurons into a phaseless set in order to achieve desynchronization (Danzl et al., 2009), or focused on the optimization of the standard HF DBS via a closed-loop stimulation setup (Feng et al., 2007a,b). In monkeys rendered parkinsonian with the neurotoxin MPTP Rosin et al. (2011) studied closed-loop DBS under acute conditions. To this end, they delivered a short train (comprising 7 pulses at 130 Hz) through a pair of electrodes located in the Globus pallidus internus (GPi) at a predetermined, fixed latency (80 ms) following each action potential recorded through an electrode placed in the primary motor cortex (M1). This type of stimulation caused a strong decrease of the firing rate of the pallidal neurons together with a pronounced decrease of the oscillatory neuronal activity at tremor frequency (4–7 Hz) and at double tremor frequency (9–15 Hz) along with an amelioration of the MPTP-induced akinesia. After cessation of this type of closed-loop DBS the initial firing pattern reverted back, i.e., pallidal firing rate and pallidal oscillatory activity attained pre-stimulus levels (Rosin et al., 2011). In contrast, standard continuous 130 Hz DBS caused a less pronounced decrease of the pallidal firing rate, the oscillatory neuronal activity and the amelioration of the akinesia (Rosin et al., 2011).

In contrast to the closed-loop DBS tested by Rosin et al. (2011), CR stimulation can be performed in a closed-loop or an open-loop mode (Tass, 2003a,b). For instance, an adaptation of the stimulation frequency to the dominant frequency of the pathological neuronal synchronized collective oscillation can increase its efficacy (Tass, 2003a,b), see also (Tass et al., 2009). However, CR stimulation is robust with respect to variations of both stimulation and model parameters as follows from both computational as well as pre-clinical and clinical studies (Tass, 2003b; Tass et al., 2012a,b). More importantly, the goal of the CR approach is fundamentally different. CR stimulation does not aim at a decrease of firing rates and/or an abolishment of oscillatory neuronal activity. Rather, CR stimulation aims at specifically counteracting pathological synchrony by desynchronization (Tass, 2003a,b). This is because neurons have to be active in order to unlearn their pathological synaptic connectivity. In this way sustained long-lasting desynchronization is induced, and, as predicted computationally (Tass and Majtanik, 2006; Hauptmann and Tass, 2007; Tass and Popovych, 2012), therapeutic effects were observed in rat hippocampal slice experiments in the context of epilepsy (Tass et al., 2009) as well as in a clinical proof of concept study in tinnitus patients treated with acoustic CR stimulation (Tass et al., 2012a), where both pathological neuronal synchrony (Adamchic et al., 2013) and pathological effective connectivity (Silchenko et al., 2013) considerably decreased. In addition, in parkinsonian (MPTP) monkeys it was shown that unilateral CR stimulation delivered to the subthalamic nucleus (STN) for only 2 h per day during 5 days leads to significant and sustained therapeutic aftereffects for at least 30 days, while standard 130 Hz DBS has no aftereffects (Tass et al., 2012b). Another motivation why CR stimulation is delivered at frequencies similar to the pathological oscillatory frequency, is that in this case the desynchronizing effect is achieved at favorably small stimulation intensities (Tass, 2003a,b). In fact, as shown computationally, CR stimulation is able to strongly alter neuronal firing rates if delivered at frequencies substantially different to the dominant frequency of the stimulated neuronal population (Lysyansky et al., 2011b). For instance, CR stimulation may effectively activate hypo- or inactive neuronal populations without inducing neuronal synchrony (Lysyansky et al., 2011b).

The CR stimulation (Tass, 2003a,b) is based on the phase reset of oscillatory neuronal activity and has a broad applicability, since the phase reset is a universal phenomenon and can be achieved for a variety of stimulation setups and conditions, see, e.g., Refs. (Winfree, 1977; Paydarfar and Eldridge, 1987; Brandt, 1997; Makeig et al., 2002; Neiman et al., 2007; Thorne et al., 2011). According to its stimulation protocol, CR stimulation counteracts synchronization in the neuronal target population by dividing the entire population into several sub-populations where the phases of the neuronal oscillators within each sub-population are reset by the stimulation sequentially, i.e., in a timely coordinated manner. In this way, the collective neuronal oscillations in the sub-populations get phase shifted with respect to each other, and the total synchronization is replaced by, e.g., a cluster state (Tass, 2003a,b; Lysyansky et al., 2011a). Due to the pathologically strong synaptic connectivity, the entire target population runs from the cluster state through a transient characterized by pronounced desynchronization and finally resynchronizes if left unperturbed. Accordingly, to keep the neuronal ensemble in a desynchronized state, CR stimuli are delivered intermittently (Tass, 2003a,b), for instance, by applying CR in an *m:n* ON-OFF mode, where a few, say *m*, cycles with CR are optimally followed by a few, say *n*, cycles without any stimulation (Lysyansky et al., 2011a), for example, with *m* = 3 and *n* = 2 (Tass et al., 2012a,b). Such a stimulation protocol has computationally been found to be effective in inducing transient desynchronization in stimulated neuronal ensembles (Tass, 2003a,b; Lysyansky et al., 2011a).

The post-stimulus transient, where the stimulation-free neurons undergo an unperturbed desynchronized dynamics, plays an important role for the emergence of long-lasting effects of CR stimulation. In computational models taking into account the adaptive synapses governed by spike timing-dependent plasticity (STDP) (Gerstner et al., 1996; Markram et al., 1997; Feldman, 2000; Wittenberg and Wang, 2006; Caporale and Dan, 2008), it has been shown that CR stimulation can lead to a reduction of the mean synaptic weight and, in turn, shift the network to a state characterized by desynchronized activity and weak connectivity (Tass and Majtanik, 2006; Hauptmann and Tass, 2007) which persists after the stimulation is switched off. Modeling shows that CR stimulation is effective for a number of stimulation setups, in particular, for direct somatic stimulation as well as for excitatory or inhibitory synaptically-meditated stimulation which corresponds to stimulation of afferent or efferent fibers (Popovych and Tass, 2012). This is particularly important since it has been shown that stimulation of fibers projecting to the STN appears to be responsible for the therapeutic effect of HF DBS delivered through STN electrodes (Gradinaru et al., 2009).

In order to further optimize the therapeutic benefit of CR stimulation, in this paper we investigate the impact of the stimulation parameters and the stimulation protocol on the stimulation-induced desynchronization. In particular, we focus on how the timing of the phase resets of the neuronal sub-populations influences the quality of the stimulation-induced cluster state and the post-stimulation transient. We found that appropriately adapted non-uniform stimulation onsets for the different stimulation sites can divide the phases of the stimulated neurons in such a way that the desynchronized post-stimulation transient gets significantly prolonged, until the population eventually resynchronizes again. To confine the complexity of our analysis, we study phase models without STDP. Phase resetting can be incorporated in such models in a natural way. We here set out to determine an optimal pattern of phase resets for CR stimulation. Put otherwise, we address the question of how to optimally choose the stimulation onsets for the single stimulation sites in CR.

In weakly coupled networks of oscillators, the technique of averaging is often applied to obtain a coupling which involves only the relative phase differences of the interacting oscillators (Ermentrout and Kopell, 1991; Swift et al., 1992; Hoppensteadt and Izhikevich, 1997). In globally coupled systems of identical neurons, these averaged phase models always possess symmetric cluster states, i.e., states, in which *m* clusters of equal size exist with a phase distance of 2π/*m* between neighbors (Okuda, 1993). Hence, a natural answer to the question above is to choose the stimulation times such that the phases get uniformly distributed over one period, independently on the type of neurons. In this case target patterns of the stimulation are the symmetric cluster states. However, in non-averaged models, the importance of the coupling between the single neurons becomes apparent. It plays an important role in determining the exact way of how the stimulation should be applied to cause a longer transient. In this work, we use systems of globally pulse-coupled phase oscillators for modeling the dynamics of a neuronal population. In particular, the symmetric cluster states disappear generically, and non-symmetric cluster states become possible candidates as stimulation target states. We propose a method for computing the stimulation times, which resets the system to a suitable cluster state. The timing points of the applied stimuli in these cases are non-uniformly spaced. The desynchronizing post-stimulation transient after such a stimulation turns out to be longer than the corresponding post-stimulation transient after a uniform CR stimulation of the same system.

The paper is organized as follows. In section 2.1 we introduce the globally pulse-coupled model that is used to describe the collective dynamics of the neurons. In section 2.2 we study the relevant dynamical properties of the model, i.e., the appearance of synchronization, symmetric clusters and splay states. CR stimulation is introduced to the model in section 2.3, and we derive how one should apply CR stimuli to obtain longer post-stimulation desynchronization transients in section 2.4. The theoretical analysis is illustrated by numerical simulations in section 3. In particular, the robustness of the results to the variation of the stimulation parameters (stimulation intensity and electrode activation time) is studied in section 3.2. The effects of inhomogeneous frequencies is studied in section 3.3.

## 2. Materials and methods

### 2.1. Pulse-coupled phase oscillators

Phase models play a key role in describing the individual dynamics of single oscillators, e.g., oscillatory neurons, see e.g., (Kuramoto, 1984). In particular, a stable periodic dynamics can be modeled by a simple equation for the periodic motion of the phase with frequency ω: φ(*t*) = φ(0)+ ωt, where φ is considered modulo 2π. The direction of the phase is neutrally stable. Therefore, a sufficiently weak temporary perturbation, which does not move the original system far away from the corresponding limit cycle, persists in the phase for all times, while all its other effects die out exponentially fast due to the stability of the limit cycle which corresponds to the periodic motion. In coupled systems, weak interactions can be conceived as perturbations, and the phase reduction can be applied as well (Kuramoto, 1984; Hansel et al., 1993; Hoppensteadt and Izhikevich, 1997; Brown et al., 2004). In fact, phase models are particularly important for studying network dynamics, because many types of synchronization, which are of interest in such models, depend on the relative phases of the units combining the network (Pikovsky et al., 2001).

**Figure 1:**
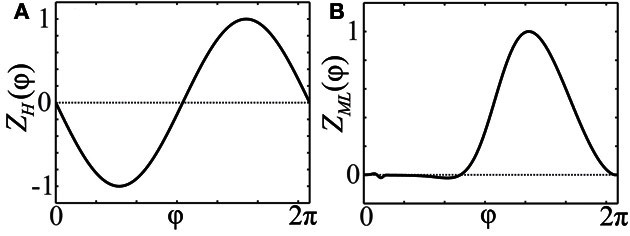
**Phase response curves. (A)** PRC *Z*(φ) = *Z*_*H*_(φ) = −sin(φ) of an oscillator close to a bifurcation of Hopf- or Bautin-type (Brown et al., 2004). **(B)** PRC *Z*(φ) = *Z*_ML_(φ) of a particular Morris-Lecar type model (Ermentrout, 1996; Sato et al., 2011).

The effect of perturbations is incorporated into the phase equations by the phase response curve (PRC) (Guckenheimer, 1975; Kuramoto, 1984; Ermentrout, 1996; Winfree, 2001) (see Figure 1). It measures the response of the individual neuron to weak stimuli. We consider the case when it admits a representation in terms of a smooth scalar function *Z*(φ) of the phase. For example, applying a weak current *I*(*t*) to a neuron with PRC *Z*(φ) changes its phase dynamics from $\dot{\varphi }(t)=\omega$ to

$$\dot{\varphi }(t)=\omega +I(t)\cdot Z(\varphi (t)),$$

which describes the phase dynamics in the weak stimulation limit (Kuramoto, 1984). If the perturbation is pulse-like, e.g., a brief electrical stimulation or a synaptic input from another neuron (if synapses are fast), it may be approximated as an instantaneous input which resets the neuron's phase at time *t* = *t*_0_ of the incoming pulse as following

$$\varphi (t_{0}^{-})\mapsto \varphi (t_{0}^{+})=\varphi (t_{0}^{-})+I\cdot Z(\varphi (t_{0}^{-})),$$

where φ(*t*^−^_0_) = lim_*t* ↑ *t*_0__φ(*t*) and φ(*t*^+^_0_) = lim_*t* ↓ *t*_0__φ(*t*). We can formally write the pulse-like perturbation as *I*(*t*) = *I* · δ (*t* − *t*_0_), using the Dirac delta-function (Goel and Ermentrout, 2002). In what follows, we study a system of *N* identical phase oscillators which are globally pulse-coupled with weight $I=\frac{\varkappa }{N}$ (Goel and Ermentrout, 2002)

(1) $${\dot{\varphi }}_{j}(t)=\omega +\frac{\varkappa }{N}Z\left(\varphi_{j}\left(t^{-}\right)\right)\sum_{k=1}^{N}\sum_{\ell }\delta \left(t_{k,\;\ell }-t\right)$$

where *t*_*k*, ℓ_ are the times where the *k*-th neuron spikes. For convenience we assume that a single neuron emits a spike if its phase crosses zero (mod 2π). In such a way, if the phase φ_*k*_ of neuron *k* passes through the spike, φ_*k*_(*t*^−^_*k*, ℓ_) = 2π, all neurons φ_*j*_ are reset to φ_*j*_(*t*^+^_*k*, ℓ_) = μ(φ_*j*_(*t*^−^_*k*, ℓ_)) where the resetting function μ is defined as

(2) $$\begin{array}{l} \mu (\varphi )=\varphi +\frac{\varkappa }{N}\cdot Z(\varphi ). \end{array}$$

In the case that *n* neurons simultaneously spike at time *t* = t_*_, the reset value of neuron φ_*j*_ is taken to be φ_*j*_(*t*^+^_*_) = μ^*n*^(φ_*j*_(*t*^−^_*_)), where μ^*n*^(φ) = μ(μ(…μ(φ)…)) denotes the *n*-fold superposition of the resetting function. This mapping is preferable to the choice of $\varphi_{j}\left(t_{*}^{+}\right)=\varphi_{j}\left(t_{*}^{-}\right)+\frac{n\varkappa }{N}Z\left(\varphi_{j}\left(t_{*}^{-}\right)\right)$ because it assures a continuous dependence on the initial conditions. More specifically, the effect of the spiking of a cluster of *n* neurons changes continuously as the neurons split from the cluster, i.e., μ(τ_1_ + μ(τ_2_ +μ(…μ(φ)…))) → μ^*n*^(φ) as the interspike intervals τ_*i*_ → 0. Note that the resetting function for the cluster spikes can also be measured or computed directly in order to achieve more realistic modeling (Achuthan and Canavier, 2009). Applied to our case it would mean using a given measured function μ(φ, *n*) instead of μ^*n*^(φ) in the case when an *n*-cluster spikes. However, we restrict our analysis to the function μ^*n*^(φ) defined as a superposition of individual resetting functions.

Note, that for sufficiently small values of ϰ/N the resetting function μ is monotone. This ensures the preservation of the ordering of the phases. We assume that *N* is sufficiently large and this property holds. For some systems (Goel and Ermentrout, 2002; Brown et al., 2004; Stiefel et al., 2009), it turned out, that the PRC has a small value at the spike moment *Z*(0) ≈ *Z*(2π) ≈ 0. For simplicity, we assume that *Z*(0) = *Z*(2π) = 0 holds for our model. Let us shortly explain why this is a reasonable approximation for weakly coupled spiking systems. Firstly, the modulus of the PRC must be roughly proportional to the density of isochrons (Guckenheimer, 1975; Winfree, 2001) at the corresponding point of the limit cycle of the full system. This density on the other hand is inversely proportional to the modulus of the vector field, which is large at the spiking point. Therefore, the modulus of the PRC has to be small at the spiking point.

Note, even though this work is focused on systems with pulse coupling (1), the main qualitative message about non-uniform CR stimulation and non-uniform positions of clusters still holds for systems of the form

(3) $${\dot{\varphi }}_{j}=\omega +\frac{\varkappa }{N}Z\left(\varphi_{j}\right)\sum_{k=1}^{N}G\left(\varphi_{k}\right),$$

with a smooth, periodic coupling function *G*, which was proposed in (Winfree, 2001). Systems (1) and (3) were less extensively studied than their averaged versions, which take the form

(4) $$\begin{array}{l} {\dot{\varphi }}_{j}=\omega +\frac{\varkappa }{N}\sum_{k=1}^{N}H\left(\varphi_{k}-\varphi_{j}\right), \end{array}$$

where *H*(φ) = (2π)^−1^∫^2π^_0_*Z*(ψ)*G*(φ + ψ)*d*ψ, see, for example, (Ermentrout and Kopell, 1991; van Vreeswijk et al., 1994; Daido, 1997; Kuramoto, 1997). As a result of the averaging, the stability properties of the corresponding solutions of (1) [or (3)] and (4) may differ at the order of $O\left(\varkappa^{2}\right)$. This is the same magnitude as of the errors made by the phase reduction, and, thus, studying the averaged system suggests itself as a simpler and, presumably, equivalent task. In the next section we show that an important genericity of stationary solutions with distributed phases is overlooked by this choice. In fact, the homogeneous stationary solutions or symmetric clusters in the averaged system (4) correspond to some other, generically non-homogeneous solutions of the original system, whose shape may differ at the order of O(ϰ), see, for instance, Equations (12) and (13) below. A precise targeting of these solutions by CR can essentially contribute to the efficacy of the desynchronization technique. Since systems (1) and (3) admit non-homogeneous stationary solutions with distributed phases, it is of particular interest to study them in the context of CR.

### 2.2. Synchronization, clusters, and stationary solutions

In this section we review the dynamical properties of the stimulation-free population (1), which are relevant in the context of desynchronization. In particular, we study the appearance and stability of a synchronized state, cluster states, as well as splay states. We pay special attention to the differences between the mentioned dynamical states of models (1), (3), and (4).

#### 2.2.1. Stability of synchronized solutions

In each of the systems (1), (3) and (4), there exists an in-phase synchronous solution where all neurons are perfectly synchronized

$$\varphi_{1}(t)=\ldots =\varphi_{N}(t).$$

The conditions for the stability of the synchronous state are well known for all of the above systems (Goel and Ermentrout, 2002). In particular, for the pulse-coupled system (1), in-phase synchronization is linearly stable iff

(5) $$\begin{array}{l} \varkappa Z^{\prime }(0)<0. \end{array}$$

For (3) the corresponding condition reads

(6) $$\begin{array}{l} \varkappa \int_{0}^{2\pi }\frac{G(\varphi )Z^{\prime }(\varphi )}{\omega +\varkappa Z(\varphi )G(\varphi )}d\varphi <0. \end{array}$$

For the averaged system (4), the stability condition of the synchronized state is

(7) $$\begin{array}{l} -\varkappa H^{\prime }(0)=\frac{\varkappa }{2\pi }\int_{0}^{2\pi }G(\varphi )Z^{\prime }(\varphi )d\varphi <0. \end{array}$$

A comparison of conditions (5)–(7) leads to the following relationships:

– Vanishing coupling: Conditions (7) and (6) for the averaged and non-averaged systems differ only in the second order of ϰ, respectively. Therefore, they coincide in the limit of small coupling ϰ → 0, if *H*′(0)≠0.
– Smooth, pulsatile coupling: If the coupling function *G*(φ) is pulse-like, i.e., it is positive and concentrated at φ = 0, then
  $$H(\varphi )\approx \bar{G}Z(-\varphi ),\bar{G}=\frac{1}{2\pi }\int_{0}^{2\pi }G(\psi )d\psi >0,$$
  and the condition for synchronization of the averaged system (7) coincides with the condition for the non-averaged pulse-coupled system (5) provided *Z*'(0) ≠ 0.

Although in this work we will focus on smooth PRCs, it is worth to mention another synchronization effect, which may generically occur for PRCs, which have discontinuous derivatives as a phase of zero is approached from the left and right (Lücken and Yanchuk, 2012): though the synchronous state is locally unstable in this case, the first order parameter $R_{1}(t)=\left|\frac{1}{N}\sum_{k}\exp\left(i\varphi_{k}(t)\right)\right|$ may still approach its maximal value *R*_1_ = 1 due to the appearance of structurally and dynamically stable homoclinic connections to the synchronous state.

#### 2.2.2. Splay states and stationary solutions

Splay states are periodic solutions of system (1), in which all oscillators are spread in a way that the time differences between the subsequent spikes *t*_*k* + 1, ℓ_−*t*_*k*, ℓ_ are always the same (Swift et al., 1992; Zillmer et al., 2007). Note that the term “splay state” can also be used differently and may, more generally, refer to any state featuring phases which are spread over the periodic interval [0, 2π] (Achuthan and Canavier, 2009). Splay states are important for desynchronization issues, since they possess small order parameters $R_{n}=\left|\frac{1}{N}\sum_{k}\exp\left(in\varphi_{k}(t)\right)\right|$.

To study splay states in large systems it is useful to consider an equation for the phase distribution density ρ(*t*, φ), since its stationary solution approximates the distribution of the phases of splay states in the limit of large *N*. For the pulse-coupled system (1) the dynamics of the phase distribution density is governed by the following continuity equation (Ernst et al., 1995; Brown et al., 2004)

(8) $$\begin{array}{l} \partial_{t}\rho (t,\varphi )=-\partial_{\varphi }\left(\rho \left(t,\varphi \right)\left(\omega +\varkappa Z(\varphi )\rho \left(t,0\right)\right)\right). \end{array}$$

Its equivalent for the smoothly-coupled model (3) is

(9) $$\begin{array}{l} \partial_{t}\rho \left(t,\varphi \right)=-\partial_{\varphi }\left(\rho \left(t,\varphi \right)\left(\omega +\varkappa Z(\varphi )\int_{0}^{2\pi }G\left(\psi \right)\rho \left(t,\psi \right)d\psi \right)\right), \end{array}$$

and for the averaged system (4) one gets

(10) $$\begin{array}{l} \partial_{t}\rho \left(t,\varphi \right)=-\partial_{\varphi }\left(\rho \left(t,\varphi \right)\left(\omega +\varkappa \int_{0}^{2\pi }H\left(\psi -\varphi \right)\rho \left(t,\psi \right)d\psi \right)\right). \end{array}$$

Solving (8) for stationary solutions ρ(*t*, φ) = ρ_*s*_(φ) and taking into account the normalization ∫^2π^_0_ρ_*s*_(ψ)*d*ψ = 1, we obtain

(11) $$\begin{array}{l} \rho_{s}(\varphi )={\left(\int_{0}^{2\pi }\frac{\omega +\varkappa Z(\varphi )\rho_{s}\left(0\right)}{\omega +\varkappa Z\left(\psi \right)\rho_{s}\left(0\right)}d\psi \right)}^{-1}. \end{array}$$

Here ρ_*s*_(0) is uniquely determined by the implicit equation obtained from (11) by inserting φ = 0 (see Appendix A.1 for details). Thus, (11) describes a unique stationary solution of (8). For small ϰ, this solution can be approximated as

(12) $$\begin{array}{l} \rho_{s}(\varphi )=\frac{1}{2\pi }+\varkappa \frac{\bar{Z}-Z(\varphi )}{{\left(2\pi \right)}^{2}\omega }+O\left(\varkappa^{2}\right), \end{array}$$

where $\bar{Z}=\frac{1}{2\pi }\int_{0}^{2\pi }Z\left(\psi \right)d\psi$ is the mean value of the PRC. For smoothly-coupled systems (3), one analogously finds that a unique stationary solution of (9) exists, if ϰ is not too large (see Appendix A.1). Its first-order expansion in ϰ reads

(13) $$\begin{array}{l} \rho_{s}(\varphi )=\frac{1}{2\pi }+\varkappa \frac{\bar{G}\left(\bar{Z}-Z(\varphi )\right)}{2\pi \omega }+O\left(\varkappa^{2}\right). \end{array}$$

For the averaged model (4), we find that for any value of ϰ the constant distribution density $\bar{\rho }(\varphi )\equiv \frac{1}{2\pi }$ is a stationary solution of (10).

**Figure 2:**
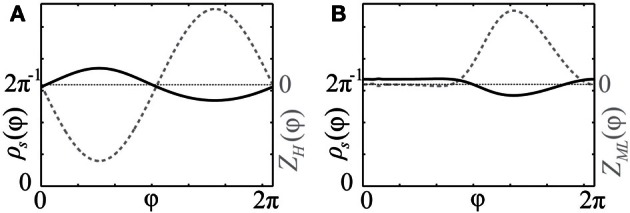
**Stationary phase distribution densities.** Black solid curves show the stationary phase distribution densities ρ_*s*_(φ) (with the scale on the left vertical axes) for the pulse-coupled systems (1) with PRCs **(A)** *Z*(φ) = *Z*_*H*_(φ) (Figure 1A) and **(B)** *Z*(φ) = *Z*_ML_(φ) (Figure 1B). The corresponding PRCs are depicted by gray dashed curves and rescaled by some constant ratio (with the scale on right vertical axes). Coupling strength ϰ = 1.0.

**Figure 3:**
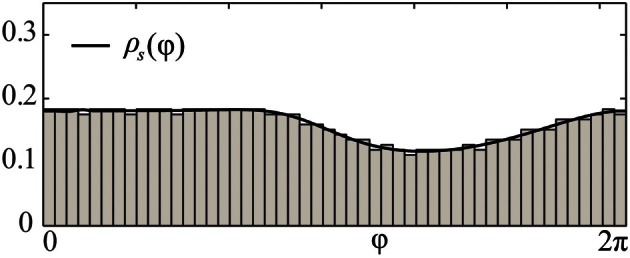
**Comparison of splay state and stationary phase distribution density.** Black solid curve depicts the theoretical solution of the stationary phase distribution density ρ_*s*_(φ) of (8) for the pulse-coupled system (1) with a PRC of Morris-Lecar type *Z*(φ) = *Z*_ML_(φ) (Figure 1B). Gray bars illustrate the numerically computed and normalized phase distribution histogram of the corresponding splay state for *N* = 1000 oscillators. Coupling strength ϰ = 3.

As follows from Equations (12) and (13), phase distributions of the splay states of the pulse-coupled system (1) as well as the non-averaged system (3) deviate from a uniform distribution. For small ϰ, the deviations can be estimated in the first order in ϰ by (12) and (13), respectively. This is in contrast to splay states of the averaged system (4), which are always uniformly distributed. Figures 2A,B illustrate non-uniform stationary phase distributions in pulse-coupled systems with PRCs *Z*_*H*_(φ) (Figure 1A) and *Z*_ML_(φ) (Figure 1B), respectively. Figure 3 shows that the theoretically obtained stationary phase distribution density ρ_*s*_(φ) (black curve) convincingly approximates the numerically calculated phase distribution histogram (gray bars) of the splay state in the pulse-coupled system (1) for large number *N* of oscillators.

**Figure 4:**
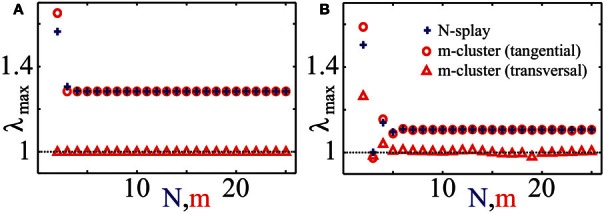
**Characteristic multipliers of splay states and cluster states.** Maximal moduli λ_max_ of characteristic multipliers of cluster states (red “◦”: tangential, red “∆”: transverse) and splay states (blue “+”) versus the number of clusters *m*, respectively the number of neurons *N*, for (A) PRC *Z* = *Z*_*H*_ and (B) PRC *Z* = *Z*_ML_. Multipliers for cluster states have been calculated asymptotically for large *N* using an asymptotic technique described in Appendix B. Coupling strength ϰ = 0.5.

The stability of splay states as well as cluster states can be studied by the set of multipliers λ of the corresponding return map, which determine the rate, with which small perturbations from the considered state are growing with time. In particular, if the multiplier with the maximal absolute value is λ_max_, then a generic perturbation will grow as λ^*j*^_max_ with *j* is the number of spikes of a neuron from the ensemble. Therefore, a stable state corresponds to the case |λ_max_| < 1 and an unstable to |λ_max_| > 1. We calculated numerically the maximal multipliers λ_max_ of the splay state of system (1) for two cases: (i) *Z*(φ) = *Z*_*H*_(φ) and (ii) *Z*(φ) = *Z*_ML_(φ) (Figure 4, blue pluses). We observe that the splay states are unstable for all *N* except *N* = 3 in case (ii), and their multipliers converge to an asymptotic value λ_∞_ > 1 as the number of neurons *N* increases. Note that the splay state may be stable for synaptic coupling and type-I PRC, as shown in (Achuthan and Canavier, 2009) for a system of a few coupled oscillators. For a more detailed analysis of the stability of splay states in large pulse-coupled systems, we refer to (Abbott and van Vreeswijk, 1993; Zillmer et al., 2007; Calamai et al., 2009).

#### 2.2.3. Symmetric clusters

In this paper we consider strongly synchronized neuronal ensembles, where the splay state is expected to be unstable. When an external desynchronization technique would be able to move the system in a vicinity of such a state, the achieved order parameter would be very low for a relatively long time. In the case, when the number of stimulation sites is naturally limited to a low number, a natural substitute for the target state of CR stimulation is a cluster state. For the sake of simplicity we will consider symmetric cluster states consisting of *m* clusters, each of them containing *N*/m neurons. Within each cluster, the neurons are synchronized and have the same phase, whereas the phases of different clusters are shifted with respect to each other. For systems (1) and (3) there exists a unique stationary, symmetric *m*-cluster state, at least for moderate coupling strength. The phases ψ_*j*_ of individual clusters are not equidistantly distributed, i.e., in general |ψ_*j* + 1_(*t*) − ψ_*j*_(*t*)| ≠ 2π/*m*. In contrast, in the averaged systems of the form (4), equidistantly distributed *m*-clusters with |ψ_*j* + 1_(*t*) − ψ_*j*_(*t*)| = 2π/*m* always exist. In Appendix B, we explain how one can determine multipliers of symmetric cluster states for system (1). We have computed them for the cases (i) *Z*(φ) = *Z*_*H*_(φ) and (ii) *Z*(φ) = *Z*_ML_(φ) (Figures 4A,B). It is convenient to distinguish between tangential and transverse multipliers, which correspond to perturbations within the invariant cluster space and perturbations which disperse the single clusters by destroying the perfect synchrony within the clusters, respectively. For case (i), and 1 ≤ *m* ≤ 25, all *m*-cluster states are unstable. In case (ii), there is a single value *m* = 3 for which the cluster state is stable. The tangential multipliers seem to asymptote towards a limit with increasing *m* (Figure 4, red circles). If the transverse multipliers are smaller than the tangential, as it is the case for both PRCs (Figure 4, red markers), one expects that the perturbed dynamics stays near the invariant cluster space as long as the linear prediction is valid. Detailed analysis of cluster states for different PRCs has been performed in (Ashwin and Swift, 1992; Hansel et al., 1993; Okuda, 1993; Chandrasekaran et al., 2011; Lücken and Yanchuk, 2012).

### 2.3. Modeling deep brain stimulation

**Figure 5:**
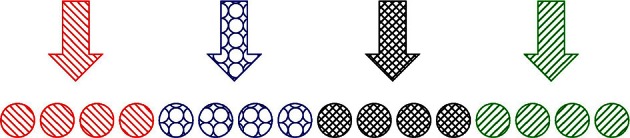
**Schematic illustration of the CR stimulation setup.** *m* = 4 stimulation sites (arrows with different filling patterns) affect the corresponding distinct sub-populations of *N*/*m* neurons (circles with the same filling pattern).

Strong enough electrical stimuli or synaptic input can reset the phase of a neuron in such a way that its oscillation restarts after the stimulation from a definite phase (Winfree, 1977; Best, 1979; Tass, 1999; Popovych and Tass, 2012). The general mechanism of phase resetting can be understood as follows. In a stimulated neuron, a stable steady (e.g., hyperpolarized) state appears, which is approached during the stimulation. When the stimulation terminates, the steady state disappears, and the system relaxes back to the limit cycle with an asymptotic phase φ_*s*_ determined by the isochron on which the stimulation specific steady state was located. However, for the phase resetting procedure which we propose, the exact value of φ_*s*_ is not essential. Therefore we include the stimulus in the simplest way which provides the model with a qualitatively adequate stimulus response. The phase model with the incorporated stimulus reads

(14) $$\begin{array}{l} {\dot{\varphi }}_{j}=\omega +Z\left(\varphi_{j}\right)\left(I_{j}(t)+\frac{\varkappa }{N}\sum_{k=1}^{N}\sum_{\ell }\delta \left(t_{k,\ell }-t\right)\right), \end{array}$$

where *I*_*j*_(*t*) corresponds to the stimulus applied to the *j*-th neuron. We assume, that there are *m* stimulation sites, each stimulating a distinct group of *N*/m neurons (Figure 5). These stimulation sites can either be active or not, that means

(15) $$\begin{array}{l} I_{j}(t)=\{\begin{array}{ll} I, & \text{ for }t\in S_{j}=\underset{i}{\cup }I_{j,i}\\ 0, & \text{else}, \end{array} \end{array}$$

where ℐ_*j*, *i*_ are stimulation time intervals and *I* is the stimulation intensity. For two neurons φ_*j*_ and φ_*k*_ from the same group we have $S_{j}=S_{k}$ and *I*_*j*_(*t*) ≡ *I*_*k*_(*t*).

The dynamics of uncoupled neurons under stimulation is described as

(16) $$\begin{array}{l} \dot{\varphi_{j}}=\omega +IZ\left(\varphi_{j}\right). \end{array}$$

**Figure 6:**
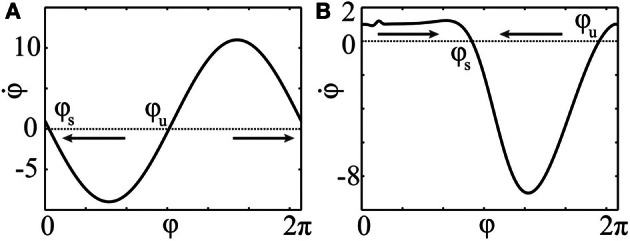
**Phase dynamics of the uncoupled neurons (16) during stimulation.** φ_*s*_ and φ_*u*_ denote stable and unstable fixed points, respectively. The arrows indicate the direction of convergence of the phase to the stable fixed point φ_*s*_. PRCs and stimulation intensities **(A)** *Z*(φ) = *Z*_*H*_(φ), *I* = 10 and **(B)** *Z*(φ) = *Z*_ML_(φ), *I* = −10.

If *IZ*(φ) < 0 for some φ, and if the intensity *I* is of sufficiently large magnitude, there appears a pair of fixed points, stable φ_*s*_ and unstable φ_*u*_, satisfying ω + *IZ*(φ) = 0. If only one such pair exists, the neuron will approach the stable fixed point φ_*s*_ after some time of stimulation and stay there until the stimulation terminates (see Figure 6). In such a situation, we call φ_*s*_ the *resetting point*.

The stimulation described by (14) aims at establishing a distribution of phases of the neuronal ensemble that prolongs the post-stimulus transient as much as possible before the ensemble synchronizes again. In principle, the strategy is to establish a state as close as possible to some stationary desynchronized state. With a given number *m* of stimulation sites which influence equally large groups of *N*/*m* neurons, the target states for the control are restricted to *m*-cluster configurations with clusters of equal size. We call a *target pattern* the state, which is intended to be realized at the end of the stimulation. A series of successive activations and deactivations of the stimulation sites is called *stimulation sequence*, and a time interval during which the resetting stimuli are delivered at all *m* sites is called *stimulation cycle*. For the averaged system (4), the target pattern consists of equidistant clusters $\psi_{j}=\frac{2\pi }{m}j+\varphi_{s}$, 1 ≤ *j* ≤ *m*, such that the last stimulation-induced cluster is located at the resetting point ψ_*m*_ = φ_*s*_ at the end of the stimulation cycle. For systems of type (1) or (3), the target pattern is a cluster state $\psi_{j}=\frac{2\pi }{m}j+\varphi_{s}+O(\varkappa )$, which is in general not equidistantly distributed. Among all possible stimulation sequences, we restrict our considerations to those where each stimulation site is activated once per stimulation cycle. The activity of the *j*-th stimulation site is confined to the time interval $S_{j}=\left[t_{j},\;t_{j}+\tau \right]$, where *t*_1_, …, *t*_*m*_ are the onset times within the stimulation cycle, and τ is the stimulation duration, which is the same for all stimulation sites. In practice, stimulation sequences have to be administered repeatedly after the system recovers to some undesired level of synchronization (Tass, 2003a).

### 2.4. Stimulation-induced stationary *M*-cluster states

Since the stimulation target pattern has to be established as precisely as possible, one has to take into account the influences of the coupling among neurons on the stimulation-induced pattern. We now describe how appropriate stimulation sequences can be found when we restrict ourselves to the stimulation timing $S_{j}=\left[t_{j},\;t_{j}+\tau \right]$, *j* = 1, …, *m*, mentioned above. The long-term desynchronizing effects of such a stimulation sequence will be discussed in the next section 3. For the brevity of notations, let us introduce the resetting map of the stimulated system (14) by

$$\begin{array}{l} \Phi :{\mathbb{R}}^{m}\times {\left[0,\;2\pi \right]}^{N}\to {\left[0,\;2\pi \right]}^{N},\\ \;(t;\varphi )\mapsto \Phi (t;\;\varphi ), \end{array}$$

where φ ∈ [0, 2π]^*N*^ is the state of the system at the onset of the stimulation at $t=t_{\text{on}}\overset{\text{def}}{=}\min\{t_{1},\;\ldots ,\;t_{m}\}$, and **t** = (*t*_1_, …, *t*_*m*_) is the vector of the onset times of the stimulation sites. Φ(**t**; φ) describes the *N*-dimensional state of the system at the end of the stimulation. If the duration τ and magnitude of the stimulation intensity *I* are large enough (see section 3.2), each neuron φ_*k*_ of the *j*-th group is reset to the collective cluster phase φ_*k*_(*t*_*j*_ + τ) ≈ φ_*s*_ at the offset time *t*_*j*_ + τ of the *j*-th stimulation site and continues to evolve in a cluster of a common phase which we denote by ψ_*j*_(*t*; φ). The dependence of ψ_*j*_ on initial conditions φ follows from the fact that this cluster may still be influenced by the other clusters and neurons. In practice, the state φ before stimulation is unknown. To ensure full control over the resulting cluster state Ψ(**t**; φ), it should be independent of the initial state φ. To this aim we have to assume that the resetting mechanism results in an accurate reset of the stimulated neurons to a determined phase φ = φ_*s*_. Furthermore, we have to ensure that the reset neurons are not affected by neurons, which have not yet been reset in the current stimulation cycle. Both conditions can be fulfilled by choosing a sufficiently large duration τ and intensity *I* for the reset. Thus, we can approximately identify the state Φ(**t**; φ) with the lower dimensional cluster state Ψ(**t**) = (ψ_1_(*t*_off_), …, ψ_*m*_(*t*_off_)), where *t*_off_ = τ + max{*t*_1_, …, *t*_*m*_} denotes the offset time of the entire stimulation sequence, and moreover, Ψ(**t**) is independent on φ. Now, let Ψ^*^ = (ψ^*^_1_, …, ψ^*^_m_) denote the phases of the stationary cluster state which serves as the stimulation target pattern and can be obtained as described in Appendix B. Then, the problem is to find a solution **t** of

(17) $$\begin{array}{l} \Psi \left(t\right)=\Psi^{*}. \end{array}$$

**Figure 7:**
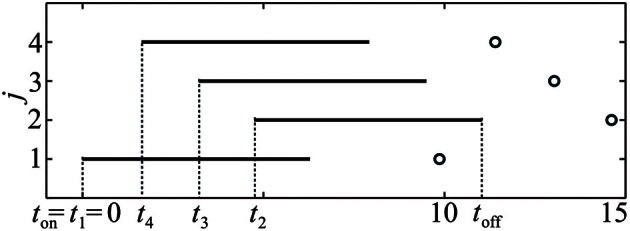
**Illustration of the stimulation sequence, which leads to the stationary 4-cluster state.** Black horizontal bars indicate time intervals when a corresponding stimulation site is active, circles mark the subsequent spike times of the established clusters. Parameter values as in Table 1.

**Figure 8:**
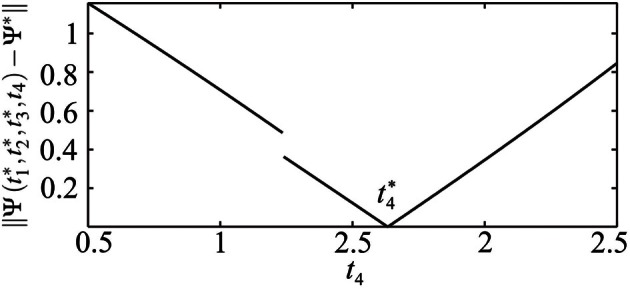
Illustration of the discontinuity of *t*_4_ ↦ Ψ(t^*^_1_, t^*^_2_, t^*^_3_, *t*_4_). ǁΨ(t^*^_1_, t^*^_2_, t^*^_3_, *t*_4_) − Ψ^*^ǁ is plotted versus *t*_4_ for fixed t^*^_1_, t^*^_2_, and t^*^_3_. Parameters as in Table 1. The discontinuity occurs at such a value of *t*_4_ = *t*_*d*_ that leads to ψ_4_(*t*^−^_off_) = 2π, i.e., the onset of the post-stimulation spike of the cluster *j* = 4 just coincides with *t*_off_, see Figure 7. If *t*_4_ > *t*_*d*_, the impact of the spike of cluster ψ_4_ on the cluster ψ_2_ has to be taken into account when calculating the resulting cluster positions.

In this study we do not aim to provide a general algorithm to solve this equation. Moreover, due to discontinuities that are caused by the pulse-like interactions of the ensemble, the function Ψ(**t**) is only piecewise smooth (see Figure 8). We approach the solution of (17) numerically by starting from the uniformly distributed stimulation sequence **t**_0_ as an initial guess. Then we apply the minimization Nelder-Mead simplex search algorithm (Lagarias et al., 1998), which is implemented in the MATLAB function fminsearch, to minimize ǁΨ(**t**) − Ψ^*^ǁ. Table 1 and Figure 7 illustrate an example of the computed stimulation sequence for the case of four stimulation sites *m* = 4 and a Morris-Lecar type of PRC *Z*(φ) = *Z*_ML_(φ) (see Figure 1). For the parameter values given in caption to Table 1, the target pattern has been computed as well as the stimulation sequence **t**. The optimal stimulation sequence deviates by up to ~4% from the uniformly distributed one where Δ t^*^_j_ is the same for all *j* = 2, …, m. Figure 7 illustrates the corresponding switching times of the stimulation contacts. It also shows the spiking times of the obtained clusters after the stimulation. In order to find the stimulation sequence, the discontinuous system (17) has been solved. Figure 8 illustrates the emerging types of discontinuities by plotting ǁΨ(t^*^_1_, t^*^_2_, t^*^_3_, *t*_4_) − Ψ^*^ǁ versus *t*_4_ for fixed t^*^_1_, t^*^_2_, and t^*^_3_.

**Table 1:** Example of target pattern (stationary cluster state) Ψ^*^ = (ψ^*^_1_, …, ψ^*^_4_) and stimulation sequence **t** = (t^*^_1_, …, t^*^_4_). Parameters: *m* = 4, *Z* = *Z*_**ML**_, ω = 1, ϰ = 0.5, τ = 2π, *I* = −10, and *t*_on_ = 0.

***j***	ψ^*^_j_	t^*^_j_	Δ t^*^_j_
1	1.169690	0	–
2	2.703008	4.749867	1.534474
3	4.284900	3.215394	1.581893
4	5.918402	1.633501	1.633501

The time elapsed between t>^*^_j_ and the preceding stimulation onset is denoted by Δ t^*^_j_. Note the non-uniformity of the stimulation. See also the corresponding illustration in Figure 7.

## 3. Results

### 3.1. Advantages to uniform stimulation

**Figure 9:**
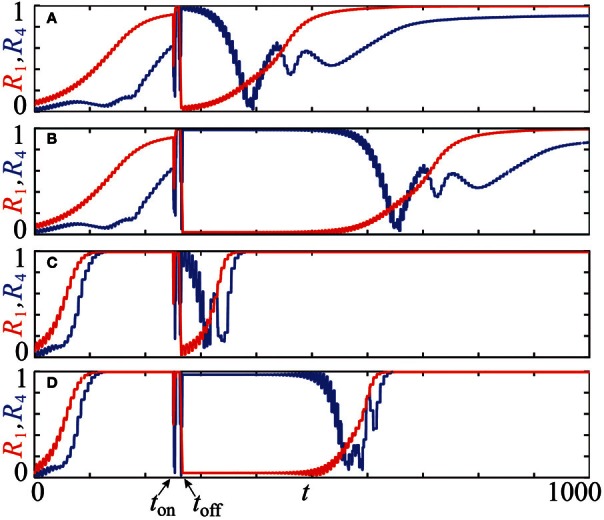
**Advantages of the stimulation-induced target patterns consisting of stationary cluster states [plots (B), (D)] compared to those induced by conventional CR stimulation with equidistant stimulation times [plots (A), (C)].** The time courses of the first order parameter *R*_1_(*t*) (red curves) and the fourth order parameter *R*_4_(*t*) (blue curves) of the neuronal ensemble (14) controlled by CR stimulation are shown for two PRCs and stimulation intensities: **(A,B)** *Z*(φ) = *Z*_ML_(φ) (Figure 1), *I* = −10 and **(C,D)** *Z*(φ) = *Z*_*H*_(φ) (Figure 1), *I* = 10. For each setup, one single CR stimulation sequence is administered at *t* = *t*_on_ = 250 with duration τ = 10 of single site activation. Number of the stimulation sites *m* = 4, system's size *N* = 240, natural frequency ω = 1, and the coupling strength ϰ = 0.5. The initial phases at *t* = 0 were randomly drawn from a uniform distribution on [0, 2π].

Equidistant clusters are stationary if the coupling depends only on the phase differences as in system (4). In the non-averaged systems (1) or (3), the phases of stationary clusters are distributed non-uniformly in [0, 2π], and the resetting technique described in section 2.4 is expected to yield longer post-stimulus transients. The results of numerical simulations of systems (1) with PRCs *Z* = *Z*_ML_ and *Z* = *Z*_*H*_ (see Figure 1) are presented in Figure 9 where plots **(A,C)** illustrate the effect of the uniform CR stimulation and **(B,D)** are related to the non-uniform CR stimulation. Time courses of the first order parameter *R*_1_(*t*) (red curves) and the fourth order parameter *R*_4_(*t*) (blue curves), defined as $R_{n}=\left|\frac{1}{N}\sum_{k}\exp\left(in\varphi_{k}(t)\right)\right|\in \left[0,\;1\right]$, are shown. Large values of the first order parameter are indicative of an in-phase synchronization. On the other hand, for approximately equidistantly distributed *m*-cluster states high values of the *m*−th order parameter are combined with low values of all order parameters with lower indices. We use these properties to detect synchronization and the discussed slightly non-uniform cluster states.

All simulations in Figure 9 are started at *t* = 0 with the neurons' phases randomly distributed in [0, 2π]. For both PRCs, *Z* = *Z*_ML_ and *Z* = *Z*_*H*_, we observe a steady increase of *R*_1_(*t*) (red curves) in the pre-stimulation epoch *t* ≲ *t*_on_, which indicates the onset of in-phase synchronization of the entire ensemble. This process is significantly faster in the case *Z* = *Z*_*H*_ (Figures 9C,D) than for *Z* = *Z*_ML_ (Figures 9A,B) due to *Z*_*H*_′(0)« *Z*_ML_′(0) ≲ 0 which strengthens the linear stability of the synchronous solution in the case *Z* = *Z*_*H*_ (see section 2.2). When CR stimulation is turned on at *t*_on_ = 250, the stimulated phases are successively caught at φ_*s*_ and released when the corresponding stimulation site is deactivated. At the end of the stimulation cycle at *t*_off_ ≈ 266.75, four clusters are established, which are well distributed in [0, 2π]. This leads to a low value of *R*_1_(*t*_off_) ≈ 0 (A: *R*_1_(*t*_off_) ≈ 0.027, B: *R*_1_(*t*_off_) ≈ 0.020, C: *R*_1_(*t*_off_) ≈ 0.000, D: *R*_1_(*t*_off_) ≈ 0.044) and a high value of *R*_4_(*t*_off_) ≈ 1 (A: *R*_4_(*t*_off_) ≈ 0.987, B: *R*_4_(*t*_off_) ≈ 0.994, C: *R*_4_(*t*_off_) ≈ 1.000, D: *R*_4_(*t*_off_) ≈ 0.969). All simulations show a post-stimulation transient before the system resynchronizes again and the first order parameter *R*_1_(*t*) approaches unity. For both considered PRCs, the advantages of the method proposed in section 2.4 to establish non-equidistant clusters is substantial. For the uniform CR stimulation (Figures 9A,C) the post-stimulation desynchronization transient is of approximately the same duration as the initial transient in the pre-stimulation epoch, when starting from a random distribution of the phases. The post-stimulation transient is significantly prolonged by the non-uniform CR stimulation in both cases, for *Z* = *Z*_ML_ (Figure 9B) by doubling the transient duration and for *Z* = *Z*_*H*_ (Figure 9D) by tripling the duration of the desynchronization transient. Note that small-scale oscillations of the order parameters originate from the discontinuities of the system's trajectory, which occur whenever a cluster crosses the firing threshold.

### 3.2. Effects of different stimulation intensities and duration

**Figure 10:**
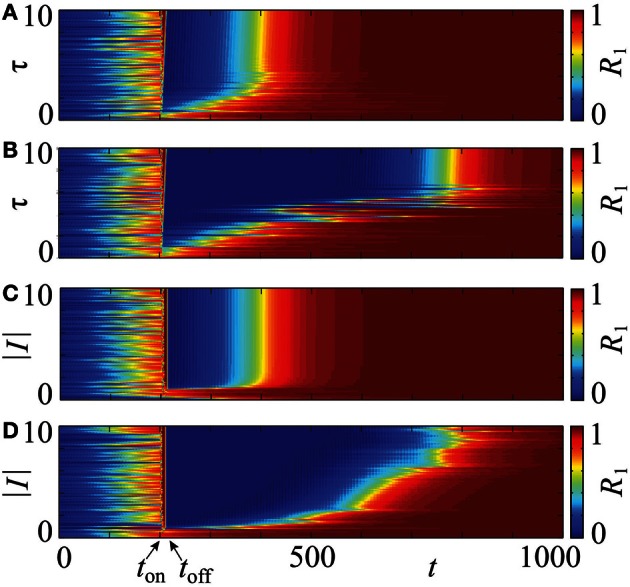
**Influence of the stimulation intensity *I* and duration τ on a post-stimulation transient in system (14) with PRC *Z* = *Z*_**ML**_.** The four charts visualize the evolution of the order parameter *R*_1_ for τ ∈ [0,10] **(A,B)**, and |*I*| ∈ [0,10] **(C,D)**. The values of the order parameter are encoded in color ranging from 0 (blue) to 1 (red), each horizontal strip of a chart corresponds to one time course as shown in Figure 9. CR stimulation is applied via *m* = 4 stimulation sites in the stimulation interval [*t*_on_, *t*_off_] with *t*_on_ = 200. The optimized non-uniform stimulation protocol is applied in **(B,D)** and the conventional, uniform stimulation timing in **(A,C)**. In **(A,B)**, *t*_off_ ranges from *t*_off_ ≈ 205 to *t*_off_ ≈ 217, and in **(C,D)**, the stimulation interval is constant. In all cases the initial phases were drawn randomly from a uniform distribution on [0, 2π]. Other parameters: ϰ = 0.5, *N* = 240.

An important question is how the stimulation intensity *I* and the stimulation duration τ influence the desynchronization transient. Figure 10 shows results of numerical simulations for the PRC *Z* = *Z*_ML_. An increase of the stimulation duration, τ ∈ [0,10], leads to an increase in the desynchronized transient equally for both, uniform (Figure 10A) and optimized, non-uniform stimulation timing (Figure 10B). This is indicated by longer intervals of decreased order parameter *R*_1_ after the stimulation between *t*_on_ and *t*_off_. For the various stimulation lengths, we have *t*_on_ = 200 and *t*_off_ ∈ [205, 217]. Beyond τ ≈ 4.5, the effect of the uniform timing does not enhance, while by the optimized protocol it increases further until about τ = τ_c_ ≈ 6.6. This value corresponds to the duration which assures independence of the stimulation outcome on the system's state prior to stimulation (see section 2.4). Similarly, small magnitudes of the stimulation intensity 1 ≲ |*I*| ≲ 2 yield a prolonged transient for both protocols (Figures 10C,D). Beyond |*I*| = 2, no further increase can be observed for the uniform timing (Figure 10C), but the transient after the non-uniform protocol continues to grow at least until |*I*| = 6 (Figure 10D).

### 3.3. Robustness to variations of natural frequencies

**Figure 11:**
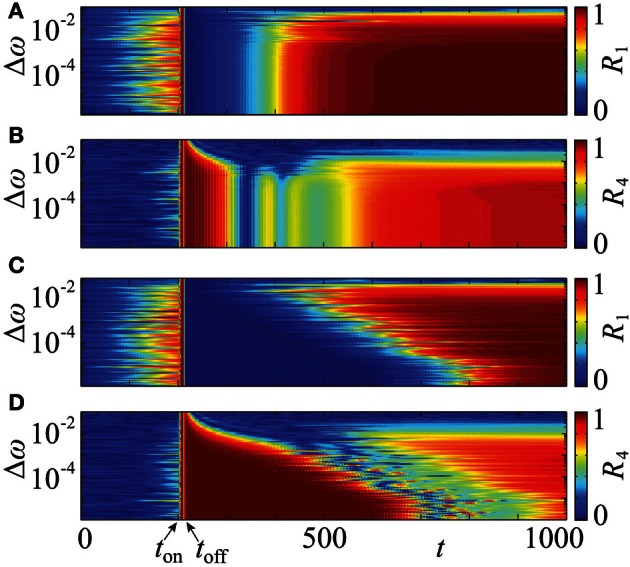
**Effect of non-identical frequencies.** Time courses of the order parameters *R*_1_ **(A,C)** and *R*_4_ **(B,D)** of the neuronal ensemble (14) for a range of the frequency detuning Δω, where the natural frequencies are randomly chosen from a uniform distribution in [1 − Δω, 1 + Δω]. The graphical representation is as in Figure 10. The conventional, uniform stimulation protocol is applied in **(A,B)** and the optimized non-uniform stimulation timing in **(C,D)**. In both cases the initial phases are randomly distributed in [0, 2π]. PRC *Z* = *Z*_ML_ (Figure 1), coupling strength ϰ = 0.5, number of oscillators *N* = 240, number of stimulation sites *m* = 4, duration of single electrode activation τ = 10, simulation onset at *t*_on_ = 200, offset at *t*_off_ ≈ 217, and stimulation strength *I* = −10.

In the above approach we assumed that the neurons are identical. In more realistic situations, the parameters of individual neurons can vary. In order to test the robustness of the proposed resetting technique with respect to parameter changes, which break the symmetry of the system, we consider an ensemble (1) with non-identical natural frequencies ω_*j*_, *j* = 1, …, *N*, e.g., randomly chosen from a uniform distribution in [1 − Δω, 1 + Δω]. The results of simulations are shown in Figure 11. We present them for the PRC *Z* = *Z*_ML_ (Figure 1), but qualitatively similar results have been obtained for *Z* = *Z*_*H*_ as well. It turns out, that a significant prolongation of the post-stimulation desynchronization transient can be observed for a range of Δω. Indeed, one observes a clear difference between the post-stimulation behavior of the order parameters *R*_1_ and *R*_4_ for the suggested CR stimulation with non-uniform stimulation timing (Figures 11C,D) and that for the conventional CR stimulation with equidistant stimulation times (Figures 11A,B). This effect of the optimized CR stimulation, however, decreases for a broader distribution of the natural frequencies. Nevertheless, our calculations suggest that the optimization procedure of CR stimulation can robustly improve its desynchronizing impact on neuronal populations exhibiting undesired synchronization.

It is well known that the broadening of the frequency range induces a desynchronizing transition (Kuramoto, 1984) such that the system with a very broad frequency range does not synchronize even in the absence of stimulation. This transition occurs at larger width of the frequency distribution if the coupling strength ϰ is increased. In our illustration with coupling strength ϰ = 0.5 (Figure 11) the desynchronization transition and the loss of the advantageous effect of the proposed stimulation technique take place at approximately the same frequency mismatch. The suggested non-uniform CR stimulation is more effective than the uniform one for a range of Δω which supports synchronized dynamics.

All results in the sections 3.2 and 3.3 hold within a range of the coupling strength, 0 < ϰ ≲ 3.5 for *Z* = *Z*_ML_ and 0 < ϰ ≲ 5.0 for *Z* = *Z*_*H*_ (data not shown). In these ranges, with larger ϰ, the re-synchronization after the stimulation happens faster, but the optimized protocol is still superior to the uniform. This means that the length of the transient following the non-uniform stimulation exceeds the one after the uniform by a similar factor as in the presented case for ϰ = 0.5. Moreover, the results are independent of the population size *N* if it is sufficiently large. In practice this is already the case for *N* ≥ 100, i.e., if each cluster contains 25 neurons.

## 4. Discussion

A number of pulsatile stimulation techniques have been developed which enable to directly shift a synchronized neuronal population into a desynchronized state, irrespective of the initial state at which the stimulus is delivered (Tass, 2001a,b, 2002a,b). However, less favorably, these techniques require careful calibration of the stimulation parameters and their continuous adaptation to varying model parameters. To overcome this limitation and provide a desynchronizing stimulation technique which is robust and does not require time-consuming or technically involved calibration procedures, CR's indirect approach to desynchronization was developed (Tass, 2003a,b): Inducing a cluster state by means of time-shifted phase resetting stimuli delivered to different neuronal sub-populations can robustly be achieved and does not require relevant calibration (Tass, 2003a,b). The cluster states, in turn, are relevant since they lead to long post-stimulus desynchronized transients (Lysyansky et al., 2011a), and in the presence of STDP (Gerstner et al., 1996; Markram et al., 1997) the related decrease of the rate of coincidences induces an anti-kindling (Tass and Majtanik, 2006; Hauptmann and Tass, 2007; Tass and Popovych, 2012). Neither in preclinical nor in clinical studies adverse effects of CR stimulation have been observed (Tass et al., 2009, 2012a,b).

We considered model networks of weakly pulse-coupled neurons with phase resetting curves and compared them to averaged models, where the phase dynamics depends only on the phase differences between the oscillators. Whereas the latter models are better analytically tractable and attained a great attention in the literature (Strogatz, 2000; Winfree, 2001; Acebrón et al., 2005), they neglect some important information about stationary states of the original systems. In particular, the stationary splay and cluster states are not uniform for the pulse-coupled networks, contrary to those of the averaged models. These non-uniformly distributed cluster states can serve as target states for CR stimulation in ensembles of pulse-coupled neurons. We have found that the optimal stimulation sequence should be slightly non-uniform in order to approach the non-uniform cluster state at the end of the stimulation.

We have shown that the phase response curves of the stimulated neurons determine the phase distribution densities of splay and cluster states, which, in turn, influence the timing of the stimulation sequences. The proposed non-uniform stimulation sequences result in significant improvements of the stimulation outcome and lead to several times longer post-stimulation transients in comparison to the equally spaced stimulation sequences. Intriguingly, modifications of the stimulation timing points of only a few percent (e.g., 4%, see Figure 7) actually double or even triple the duration of the post-stimulation desynchronization transient (Figure 9). The proposed approach takes into account and compensates for the interactions among neurons during the stimulation.

We also showed that the discussed stimulation protocol is robust with respect to variation of the natural frequencies, stimulation parameters, and coupling strength. It can lead to a prolonged transient for a range of non-identical frequencies of the single oscillators. One can expect that the non-uniform stimulation technique can be superior to a series of equally timed stimulations in more diverse and realistic setups, where transmission delays and coupling functions are heterogeneous and this should be confirmed in further studies. Moreover, since the mechanism of the discussed desynchronizing method is based on the phase reset of the neuronal oscillations, which is a universal phenomenon of the neuronal dynamics (Winfree, 1977; Best, 1979; Tass, 1999), and the timing of the optimal stimulation sequence is determined by the phase response curves, the presented approach is generic and can be applied to other neuronal models and other stimulation-induced target states. In particular, to models, which employ PRCs of second, or higher order (Oprisan et al., 2004; Achuthan and Canavier, 2009). For our purpose, we restricted the investigation to the system (1), since it is one of the simplest models, which already possesses enough features illustrating our main finding, that the optimal stimulation timing is non-uniform. In the framework of the considered model one can, however, incorporate the PRCs measured either experimentally or obtained by detailed modeling of the globus pallidus and STN (Schultheiss et al., 2010; Farries and Wilson, 2012b,a) which are possible target regions for CR deep brain stimulation (Popovych and Tass, 2012; Tass et al., 2012b), and where a change in PRC structure might contribute to disease-related changes in synchronous activity. Another target for non-invasive acoustic CR neuromodulation adapted for the treatment of tinnitus (Tass and Popovych, 2012; Tass et al., 2012a) is the auditory cortex where a phase reset can be achieved by different types of auditory stimuli (Brandt, 1997; Thorne et al., 2011).

As yet, only slight modifications of the τ/*m* timing of CR stimulation (with onset times *t*_1_, *t*_1_ + τ/*m*, *t*_1_ + 2τ/*m*, …, *t*_1_ + (*m* − 1)τ/*m*) have been investigated in the Kuramoto model as well as in an ensemble of synaptically coupled FitzHugh–Nagumo oscillators modeling spiking neurons in the context of *M:N* ON-OFF CR stimulation, where *M* cycles with CR are followed by *N* cycles without stimulation (Lysyansky et al., 2011a). For that stimulation protocol a τ/*m* timing of CR stimulation was used. However, in one variant of this protocol the *M*-th stimulation cycle was prematurely terminated at the break time *t*_off_, where *t*_off_ < τ. An optimal choice of *t*_off_ (e.g., *t*_off_ = 0.44τ for a particular set of model parameters tested) caused a pronounced increase of the desynchronization transient, i.e., an increase of the time elapsing to resynchronization by a factor of approx. 2. In contrast, inappropriate values of *t*_off_ induced a decrease (e.g., a halving) of the resynchronization time.

To study post-stimulus desynchronization transients of two phase oscillators with time-delayed coupling subject to coincident, but phase shifted stimulation the transient time *T*_tr_ (defined as the time it takes a trajectory after stimulus offset to permanently enter into an ε vicinity of the stable phase-locked state) was computed (Krachkovskyi et al., 2006). For vanishing time delays the phase space of that two-oscillator model is simple and the optimal phase shift in the coupling term puts the system's phase difference onto an unstable fixed point (Figure 9a in Krachkovskyi et al., 2006). In contrast, for non-vanishing time delay the phase space gets considerably more complex, and the optimal phase shift puts the system onto a particular point in phase space where the system gets trapped by a stable manifold, leading to a particularly high transient time *T*_tr_. Incorporating time delays into the coupling of the model studied in this paper will certainly increase the complexity of its phase space. Given the results by Ref. (Krachkovskyi et al., 2006), we expect that such timed delays may have an impact on the resynchronization transient and, hence, on the optimal timing of CR stimulation.

Another important direction for future analysis comes from the fact that biological networks typically comprise neurons of different kind. Consequently, our approach has to be extended to mixed populations, containing neurons of different type by, for example, including inhibitory neurons found in human STN (Levesque and Parent, 2005). In this work we considered a simple all-to-all coupling topology which provides an easy way to obtain a synchronized neuronal dynamics serving as a model for the dynamical regimes encountered in Parkinsonian patients and monkeys (Nini et al., 1995; Levy et al., 2000). This was also motivated by the reported strong functional connectivity of tremor-related neurons in STN (Amtage et al., 2009) and anatomical intranuclear connectivity as follows from experimental and modeling studies (Iwahori, 1978; Kita et al., 1983; Gillies and Willshaw, 1998, 2004; Shen and Johnson, 2006), but see Ref. (Wilson et al., 2004). The considered weak coupling is supported by the observed gradual decay and recovery of pathological oscillations at the onset and offset of DBS (Kang and Lowery, 2011). CR stimulation has been shown to work for other coupling topologies and stimulation setups, e.g., for sensory stimulation (Popovych and Tass, 2012; Tass and Popovych, 2012; Tass et al., 2012a). For further details of the effects of CR stimulation, more realistic coupling topologies and sophisticated neuronal models as well as connections to other neural populations within the basal ganglia and cortical brain areas have to be considered. Also, the spatial spread of the stimulation current, 3D effects as well as optimized electrode geometries (see e.g., Buhlmann et al., 2011) have to be taken into account in future studies. Pursuing such studies, our ultimate goal is to come up with CR sequences which enable to further minimize the stimulation current for DBS. This might contribute to a decrease of the rate of side effects caused by stimulation spread to neighboring brain areas. By the same token, this might enable considerably smaller geometries of the implantable pulse generator due to a significant reduction of battery size.

### Conflict of interest statement

The authors declare that the research was conducted in the absence of any commercial or financial relationships that could be construed as a potential conflict of interest.

## Appendix

### A. Stationary solutions of the phase density equations

#### A.1 Pulse coupled systems

In this section we show how a stationary solution ρ(*t*, φ) = ρ_*s*_(φ) for (8) can be found and show that it is unique. It follows from (8) that this solution satisfies

(A1) $$\rho_{s}(\varphi )\left(\omega +\varkappa Z(\varphi )\rho_{s}\left(0\right)\right)=C$$

with some constant *C*. Hence

$$\rho_{s}(\varphi )=\frac{C}{\omega +\varkappa Z(\varphi )\rho_{s}\left(0\right)}.$$

The constant *C* can be determined from the normalization condition

(A2) $$\begin{array}{l} \int_{0}^{2\pi }\frac{C}{\omega +\varkappa Z\left(\psi \right)\rho_{s}\left(0\right)}d\psi =1. \end{array}$$

Combining (A1) and (A2), we obtain the expression (11) for ρ_*s*_(φ). Evaluating (11) at φ = 0 and taking into account *Z*(0) = 0, the equation for ρ_*s*_(0) reads

$$\rho_{s}\left(0\right)={\left(\int_{0}^{2\pi }\frac{d\psi }{1+\frac{\varkappa }{\omega }Z\left(\psi \right)\rho_{s}\left(0\right)}\right)}^{-1}.$$

To investigate the resolvability of this equation we consider the function

$$F\left(x\right):={\left(\int_{0}^{2\pi }\frac{d\psi }{1+\frac{\varkappa }{\omega }Z\left(\psi \right)x}\right)}^{-1}-x,$$

which is well-defined and smooth on *x* ∈ (0, ζ) with ζ = ∞ in case ϰ *Z*(φ) ≥ 0, and $\zeta =\frac{-\omega }{\min_{\psi }\varkappa Z\left(\psi \right)}$ otherwise. It is easy to see that $F\left(0\right)=\frac{1}{2\pi }>0$. Furthermore, we will show that

(i) lim_*x* → ζ_ *F*(*x*) = −ζ < 0 and
(ii) *F*″(*x*) ≤ 0.

The conditions (i) and (ii) imply that *F*(*x*) has a unique root, which corresponds to a unique solution ρ_*s*_(φ) of (8). In order to show (i), we treat two cases separately. First, if ζ < ∞, then the smoothness of *Z*(ψ) leads to

$$\underset{x\to \zeta }{\lim}\left(\int_{0}^{2\pi }\frac{d\psi }{1+\frac{\varkappa }{\omega }Z\left(\psi \right)x}\right)=\infty$$

and, therefore, lim_*x* → ζ_ *F*(*x*) = −ζ. In the second case, when ζ = ∞, we use the following estimates: Since *Z*(ψ) is smooth and *Z*(0) = 0, one can always find a large enough constant *L* > 0 such that

$$\frac{\varkappa }{\omega }Z\left(\psi \right)\leq L\tilde{Z}\left(\psi \right),\tilde{Z}\left(\psi \right)=\{\begin{array}{ll} \psi , & 0\leq \psi \leq \pi \\ 2\pi -\psi , & \pi \leq \psi \leq 2\pi . \end{array}$$

Now we estimate *F*(*x*) as follows

$$\begin{array}{l} F\left(x\right)\leq {\left(\int_{0}^{2\pi }\frac{d\psi }{1+L\tilde{Z}\left(\psi \right)x}\right)}^{-1}-x\\ \;=\frac{Lx}{2\ln\left(1+L\pi x\right)}-x. \end{array}$$

Hence lim_x → ∞_*F*(*x*) = −∞.

Now let us show that (ii) holds. The second derivative of *F*(*x*) can directly be calculated and reads

$$F^{\prime \prime }(x)=2{\left(\int_{0}^{2\pi }gd\psi \right)}^{-3}F_{1}(x),$$

where $g\left(\psi ,\;x\right)={\left(1+\frac{\varkappa }{\omega }Z\left(\psi \right)x\right)}^{-1}>0$, $h\left(\psi \right)=\frac{\varkappa }{\omega }Z\left(\psi \right)$, and

$$F_{1}={\left(\int_{0}^{2\pi }g^{2}fd\psi \right)}^{2}-\int_{0}^{2\pi }g^{3}f^{2}d\psi \int_{0}^{2\pi }gd\psi .$$

The sign of *F*″ coincides with the sign of *F*_1_. We apply the Cauchy-Schwarz's inequality to obtain

$$\begin{array}{l} \;{\left(\int_{0}^{2\pi }g^{2}fd\psi \right)}^{2}={\langle g^{\frac{1}{2}},g^{\frac{3}{2}}f\rangle }_{L^{2}}^{2}\\ \leq {\left\|g^{\frac{1}{2}}\right\|}_{L^{2}}{\left\|g^{\frac{3}{2}}f\right\|}_{L^{2}}=\int_{0}^{2\pi }g^{3}f^{2}d\psi \int_{0}^{2\pi }gd\psi . \end{array}$$

Thus, *F*_1_ ≤ 0 holds and claim (ii) is proven.

We have shown that the equation for the phase density for the pulse-coupled system (8) has a unique stationary solution. Note that smallness of ϰ was not assumed here. The first order approximation in ϰ of this density takes the form (12).

#### A.2 Smoothly coupled systems (3) and their averaged counterpart (4)

To unify notations, we write the dynamics for the single neurons in the limit of large population as

$$\dot{\varphi }=\omega +\varkappa K\left(\varphi ,\rho \left(t,\cdot \right)\right),$$

where

$$K(\varphi ,\rho (t,\cdot ))=\{\begin{array}{ll} Z(\varphi )\overset{2\pi }{\underset{0}{\int }}G(\xi )\rho (t,\xi )d\xi , & for\;(3),\\ \overset{2\pi }{\underset{0}{\int }}H(\varphi -\xi )\rho (t,\xi )d\xi , & for\;(4). \end{array}$$

The phase density equation reads

$$\partial_{t}\rho (t,\varphi )=-\partial_{\varphi }\left[\rho \left(t,\varphi \right)\left(\omega +\varkappa K\left(\varphi ,\rho_{s}\left(\cdot \right)\right)\right)\right]$$

and its stationary solution ρ_*s*_(φ) fulfills

(A3) $$\begin{array}{lll} C & = & \rho_{s}(\varphi )\left(\omega +\varkappa K\left(\varphi ,\rho_{s}\left(\cdot \right)\right)\right) \end{array}$$

with some constant *C* depending on ρ_*s*_(·). Solving for ρ_*s*_(φ) and integration over [0, 2π] with respect to φ gives an expression for *C*

$$C={\left(\int_{0}^{2\pi }\frac{d\psi }{\omega +\varkappa K\left(\psi ,\rho_{s}\left(\cdot \right)\right)}\right)}^{-1}.$$

Substitution back into (A3) yields

(A4) $$\begin{array}{lll} \rho_{s}(\varphi ) & = & {\left[\int_{0}^{2\pi }\frac{\omega +\varkappa K\left(\varphi ,\rho_{s}\left(\cdot \right)\right)}{\omega +\varkappa K\left(\psi ,\rho_{s}\left(\cdot \right)\right)}d\psi \right]}^{-1}\\ & = & \frac{1}{2\pi }+O\left(\varkappa \right). \end{array}$$

We see that the stationary solution is generally a ϰ-perturbation of the constant state. Without going into details, we remark that for small ϰ the right hand side of (A4) is a contraction with respect to ρ_*s*_(·) on all sets ℳ_β_ = {*f* ∈ *C*([0, 2π]) | *f* ≥ 0, ǁ*f*ǁ_1_ = 1, ǁ*f*ǁ_∞_ ≤ β} of all continuous densities on [0, 2π] which are bounded by some $\beta >\frac{1}{2\pi }$. This implies that there is a unique stationary solution in this set, which can be computed by iteration.

### B. Stability of symmetric cluster states

In this section, we present a method for calculating characteristic multipliers of the symmetric cluster states of system (1) (see section 2.2.3). To this end, we introduce a discrete time map (B1) which describes the system's evolution in between two subsequent spikes. The main idea of our analysis is a separation of the effects of tangential and transversal perturbations to the cluster state. This allows us to derive the expressions (B3) and (B5) for the characteristic multipliers which can be evaluated easily. Finally, we explain how these multipliers behave asymptotically as *N* → ∞.

Let us consider a time moment *t*_0_, when some neuron of the ensemble (1) just emitted a spike. We choose its index to be *N* and all other indices in such a way, that the phases are ordered as 2π ≥ φ_1_(*t*^+^_0_) ≥ … ≥ φ_*N*_(*t*^+^_0_) = 0. Until the next spiking event, all neurons advance with the same phase velocity ω. Without loss of generality, we can assume ω = 1. Then the next neuron φ_1_ reaches the spiking threshold φ_1_(*t*^−^_1_) = 2π at time *t*_1_ = *t*_0_ + 2 π − φ_1_(*t*^+^_0_). The other neurons φ_*k*_ are now located at φ_*k*_(*t*^−^_1_) = φ_*k*_(*t*^+^_0_) + 2π − φ_1_(*t*^+^_0_). After the spike, the first neuron is reset to φ_1_(*t*^+^_1_) = 0 and all others to φ_*k*_(*t*^+^_1_) = μ(φ_*k*_(*t*^−^_1_)) = μ(φ_*k*_(*t*^+^_0_) + 2π − φ_1_(*t*^+^_0_)). Because of the monotonicity of the resetting map μ, the order of the phases is preserved as 2π ≥ φ_2_(*t*^+^_1_) ≥ … ≥ φ_*N*_(*t*^+^_1_) ≥ φ_1_(*t*^+^_1_) = 0. We define the firing map as $F:\;T^{N}\to T^{N}$, T=[0, 2π]/{0~2π} (the points 0 and 2π are identified as the same point) componentwise by

(B1) $$F_{k}(\varphi ):=\mu \left(\varphi_{k+1}+2\pi -\varphi_{1}\right),\;1\leq k\leq N-1,$$

and *F*_*N*_(φ): = 0. It captures the threshold crossing and spiking of the oscillator φ_1_ and shifts the indices *k* ↦ *k* − 1. This map takes the ordered phases after some spiking event 2π ≥ φ_1_ ≥ … ≥ φ_*N*_ = 0 and maps them to ordered phases after the successive spiking event 2π ≥ *F*_1_(φ) ≥ … ≥ *F*_*N*_(φ) = 0. Since we assume the resetting function μ(φ) to be smooth on T, *F* is also smooth on $T^{N}$.

The *N*-fold iteration of the firing map *R*: = *F*◦ … ◦ *F* = *F*^*N*^ is called the return map. Given an initial state φ with φ_1_ ≥ … ≥ φ_*N*_, the map *R*(φ) returns the state after each neuron has fired once. In order to simplify notations, we adopt double indices, φ_ℓ, *j*_: = φ_(ℓ − 1)*n* + *j*_, to address the *j*-th member of the ℓ-th cluster. A stationary, symmetric *m*-cluster state is a fixed point φ^*^ = *F*^*n*^(φ^*^) for the *n* = *N*/*m*-th iterate of the firing map, which satisfies φ^*^_ℓ, *j*_ = ψ_ℓ_ with 1≤ ℓ ≤ *m*, 1 ≤ *j* ≤ *n*, and ordered cluster positions 2π > ψ_1_ > … > ψ_*m*_ = 0. For such a symmetric cluster state, the time τ elapsed between two successive threshold crossings of clusters is the same. This time τ can be determined as the smallest solution of the equation *G*^*m* − 1^_τ_(0) + τ = 2π. Here the function *G*_τ_(*x*): = μ^*n*^(*x* + τ) describes the phase resetting of a neuron located at position *x* + τ induced by a cluster spike. The value of *G*^*m* − 1^_τ_(0) = *G*_τ_◦ *G*_τ_◦ … ◦ *G*_τ_(0) is the position of a cluster initially located at ψ = 0 after *m*−1 other clusters have fired. The positions of other clusters are then given as ψ_ℓ_ = *G*^*m* − ℓ^_τ_(0). The linear stability of the cluster state φ^*^ can be determined by its characteristic multipliers with respect to *R*, i.e., the eigenvalues of *DR*(φ^*^). In the following we calculate these multipliers. Using double indices for *F* in the same way as for φ, we have

$$F_{\ell ,j}^{n}\left(\varphi^{*}\right)=\mu^{n}\left(\psi_{\ell +1}+2\pi -\psi_{1}\right)=\psi_{\ell },$$

where the index ℓ + 1 is considered modulo *m* within the range from 1 to *m*. For a perturbed cluster state, the phase of each neuron can be written as φ_ℓ, *j*_ = ψ_*j*_ + η_ℓ, *j*_. To study the dynamics of the perturbations, we introduce the following subspaces

$$\begin{array}{l} W=\overset{m}{\underset{k=1}{\cap }}\{\eta_{k,j}=\eta_{k,i},\;for\;1\leq i,j\leq n\},\\ V_{\ell }=\underset{k\neq \ell }{\cap }\{\eta_{k,j}=0,\;for\;1\leq j\leq n\}\cap^{\text{​}}\{\eta_{\ell ,n}=0\}, \end{array}$$

1 ≤ ℓ ≤ *m*. Elements of W correspond to perturbations, which rule the relative motions of the clusters. Correspondingly, the subspace $V_{\ell }$ contain perturbations, which split the ℓ-th cluster. Since the perturbations from $V_{\ell }$ cannot split any other clusters, we have

$$F^{n}\left(\varphi^{*}+\eta \right)\in \{\begin{array}{ll} \varphi^{*}+W & \text{ for }\eta \in W,\\ \varphi^{*}+V_{\ell -1}⊕W & \text{ for }\eta \in V_{\ell }. \end{array}$$

This implies

(B2) $$DF^{n}\left(\varphi^{*}\right)V_{\ell }\subset V_{\ell -1}⊕W,\text{ and }DF^{n}\left(\varphi^{*}\right)W\subset W.$$

By *m* applications of the map *DF*^*n*^ = *DF*^*n*^(φ^*^), we obtain the following sequence

$$V_{\ell }\overset{DF^{n}}{\to }V_{\ell -1}⊕W\overset{DF^{n}}{\to }\cdots \overset{DF^{n}}{\to }V_{\ell -m}⊕W=V_{\ell }⊕W.$$

Hence, for *DR*(φ^*^) = (*DF*^*n*^(φ^*^))^*m*^, we have the skew structure $DR\left(\varphi^{*}\right)V_{\ell }\subset W⊕V_{\ell }$ and $DR\left(\varphi^{*}\right)W\subset W$. This implies that the matrix *DR*(φ^*^) has a block triangular form in an appropriate basis and the spectrum of eigenvalues of *DR*(φ^*^) is the union of the spectra, which are restricted to the subspaces W and $V_{\ell }$. The eigenvalues belonging to the subspace $V_{\ell }$, can be found by considering all possible perturbations in $V_{\ell }$. These perturbations can be described as a linear combination of the following basis vectors

$$\eta_{k,j}^{i}=\{\begin{array}{ll} 1, & \text{for }k=\ell ,1\leq j\leq i,\\ 0, & \text{else, } \end{array}$$

for 1 ≤ *i* ≤ *n* − 1. Each element η^*i*^ corresponds to a different way to split the ℓ-th cluster into two subclusters. It is possible to show that the multiplier corresponding to η^*i*^ is given as the change of the distance of these two subclusters during one return. Calculating the perturbed dynamics step by step (from one cluster firing to the next) and linearizing in η^*i*^ leads to

$$DR\left(\varphi^{*}\right)\eta^{i}=\lambda \eta^{i}+w$$

with some w∈W and the eigenvalue λ independent on ℓ and *i*.

(B3) $$\lambda =\prod_{\ell =1}^{m}\delta_{\ell },\;\delta_{\ell }={\left(\mu^{n}\right)}^{\prime }\left(\psi_{\ell }+2\pi -\psi_{1}\right).$$

The perturbations in W can be described by the following basis vectors

$$\eta_{k,j}^{\ell }=\{\begin{array}{ll} 1, & \text{for }k=\ell ,1\leq j\leq n\\ 0, & \text{else.} \end{array}$$

Each element η^ ℓ^ corresponds to a perturbation of the ℓ-th cluster, which does not split the cluster. Calculations (for brevity, we omit the details here) of the dynamics of η^ ℓ^ show that the eigenvalues of *DR*(φ^*^) restricted to W are given as the *m*-th powers of the eigenvalues of the matrix

(B4) $$\begin{array}{l} A=\left[\begin{array}{cccc} -\delta_{2} & \delta_{2} & 0 & 0\\ \vdots & 0 & \ddots & 0\\ -\delta_{m} & \vdots & \ddots & \delta_{m}\\ 0 & 0 & \cdots & 0 \end{array}\right]. \end{array}$$

This means that tangential multipliers of the symmetric *m*-cluster state are given as λ^*m*^ where λ are the solutions of

(B5) det(A−λI)=0.

As explained in detail in (Lücken and Yanchuk, 2012), the repetitive firing $\mu^{n}(\varphi )\approx \vartheta (\frac{1}{m},\;\varphi )$ can be approximated by the solution $\vartheta (\frac{1}{m},\;\varphi )$ of the initial value problem

(B6) $$\frac{d\vartheta }{dr}\left(r,\varphi \right)=\varkappa Z\left(\vartheta \left(r,\varphi \right)\right),\text{ with }\vartheta \left(0,\varphi \right)=\varphi .$$

Furthermore, we have

(B7) $$\begin{array}{l} \frac{d\vartheta }{d\varphi }\left(\frac{1}{m},\varphi \right)=\{\begin{array}{ll} \frac{Z\left(\vartheta \left(\frac{1}{m},\varphi \right)\right)}{Z(\varphi )}, & \text{for }Z(\varphi )\neq 0,\\ \exp\left(\frac{\varkappa }{m}Z^{\prime }(\varphi )\right), & \text{for }Z(\varphi )=0. \end{array} \end{array}$$

This gives an asymptotic method to determine position and stability of *m*-clusters in the limit *N* → ∞ by determining the quantities ${\bar{\delta }}_{\ell }=\frac{d\vartheta }{d\varphi }\left(\frac{1}{m},\;{\bar{\psi }}_{\ell }\;+\;2\pi \;-\;{\bar{\psi }}_{1}\right)$ for the fixed point $\bar{\psi }=\left({\bar{\psi }}_{1},\;\ldots ,\;{\bar{\psi }}_{m}\right),\;{\bar{\psi }}_{1}>\;\cdots \;>\;{\bar{\psi }}_{m}=0$, of the asymptotic *m*-cluster map ${\bar{F}}_{m}:\;T^{m}\to T^{m}$, which is defined componentwise as

(B8) $${\bar{F}}_{m,\ell }\left(\psi \right)=\vartheta \left(\frac{1}{m},\psi_{k+1}+2\pi -\psi_{1}\right),\;1\leq \ell \leq m.$$

This fixed point approximates the stationary, symmetric *m*-cluster φ^*^ in the limit of large *N*. The stability of φ^*^ can be determined by replacing δ_ℓ_ with ${\bar{\delta }}_{\ell }$ in the expressions (B3) and (B4). The asymptotic cluster positions $\bar{\psi }$ can be determined from the smallest solution τ of the equation ${\bar{G}}_{\tau }^{m-1}\left(0\right)+\tau =2\pi$, where ${\bar{G}}_{\tau }(\varphi )=\vartheta \left(\frac{1}{m},\;\varphi \;+\;\tau \right)$.

### C. Model equations

The dimensionless Morris-Lecar neuron model (Ermentrout, 1996; Sato et al., 2011) is given by the following equations

$$\begin{array}{l} \dot{V}=I-g_{L}(V-V_{L})-g_{K}w(V-V_{K})\\ \;-g_{Ca}m_{\infty }(V)(V-V_{Ca}),\\ \dot{w}=\mu \lambda (V)(w_{\infty }-w), \end{array}$$

with

$$\begin{array}{l} m_{\infty }(V)=\frac{1}{2}(1+\tanh(\frac{V-V_{1}}{V_{2}})),\\ w_{\infty }(V)=\frac{1}{2}(1+\tanh(\frac{V-V_{3}}{V_{4}})),\\ \;\lambda (V)=\frac{1}{3}\cosh(\frac{V-V_{3}}{2V_{4}}). \end{array}$$

Parameters have been chosen according to references (Ermentrout, 1996; Sato et al., 2011) as *V*_*L*_ = −0.5, *V*_*K*_ = −0.7, *V*_Ca_ = 1.0, *g*_*L*_ = 0.5, *g*_*K*_ = 2, *g*_Ca_ = 1.33, *V*_1_ = −0.01, *V*_2_ = 0.15, *V*_3_ = 0.1, *V*_4_ = 0.145, *I* = 0.0695 and μ = 0.25. We have obtained the PRC *Z*(φ) = *Z*_ML_(φ) for perturbations *V* ↦ *V* + Δ *V* by direct simulation for Δ*V* = 0.0025 and setting $Z_{\text{ML}}(\varphi )=-\frac{2\pi \Delta T}{T\Delta V}$, where *T* is the unperturbed period of the model and Δ*T* is the asymptotic phase lag caused by the perturbation.

## References

[B1] Abbott L. F. van Vreeswijk C. (1993). Asynchronous states in networks of pulse-coupled oscillators. Phys. Rev. E 48, 1483–1490 10.1103/PhysRevE.48.1483 9960738

[B2] Acebrón J. A. Bonilla L. L. Vicente C. J. P. Ritort F. Spigler R. (2005). The Kuramoto model: a simple paradigm for synchronization phenomena. Rev. Mod. Phys. 77, 137–185

[B3] Achuthan S. Canavier C. (2009). Phase-resetting curves determine synchronization, phase locking, and clustering in networks of neural oscillators. J. Neurosci. 29, 5218–5233 10.1523/JNEUROSCI.0426-09.2009 19386918 PMC2765798

[B4] Adamchic I. Toth T. Hauptmann Ch. Tass P. A. (2013). Reversing pathologically increased electroencephalogram power by acoustic coordinated reset neuromodulation. Hum. Brain Mapp. (in press). 10.1002/hbm.22314 PMC4216412 23907785

[B5] Amtage F. Henschel K. Schelter B. Vesper J. Timmer J. Lucking C. H. (2009). High functional connectivity of tremor related subthalamic neurons in parkinson's disease. Clin. Neurophysiol. 120, 1755–1761 10.1016/j.clinph.2009.06.018 19632151

[B6] Ashwin P. Swift J. W. (1992). The dynamics of n weakly coupled identical oscillators. J. Nonlin. Sci. 2, 69–108

[B7] Benabid A. L. Pollak P. Gervason C. Hoffmann D. Gao D. M. Hommel M. (1991). Longterm suppression of tremor by chronic stimulation of ventral intermediate thalamic nucleus. Lancet 337, 403–406 1671433 10.1016/0140-6736(91)91175-t

[B8] Best E. N. (1979). Null space in the Hodgkin-Huxley equations. A critical test. Biophys. J. 27, 87–104 10.1016/S0006-3495(79)85204-2 262379 PMC1328549

[B9] Brandt M. E. (1997). Visual and auditory evoked phase resetting of the alpha EEG. Int. J. Psychophysiol. 26, 285–298 9203010 10.1016/s0167-8760(97)00771-x

[B10] Brown E. Moehlis J. Holmes P. (2004). On the phase reduction and response dynamics of neural oscillator populations. Neural Comput. 16, 673–715 10.1162/089976604322860668 15025826

[B11] Buhlmann J. Hofmann L. Tass P. A. Hauptmann C. (2011). Modeling of a segmented electrode for desynchronizing deep brain stimulation. Front. Neuroeng. 4:15 10.3389/fneng.2011.00015 22163220 PMC3233722

[B12] Calamai M. Politi A. Torcini A. (2009). Stability of splay states in globally coupled rotators. Phys. Rev. E 80:036209 10.1103/PhysRevE.80.036209 19905202

[B13] Caporale N. Dan Y. (2008). Spike timing-dependent plasticity: a hebbian learning rule. Annu. Rev. Neurosci. 31, 25–46 10.1146/annurev.neuro.31.060407.125639 18275283

[B14] Chandrasekaran L. Achuthan S. Canavier C. C. (2011). Stability of two cluster solutions in pulse coupled networks of neural oscillators. J. Comput. Neurosci. 30, 427–445 10.1007/s10827-010-0268-x 20725773 PMC3059341

[B15] Daido H. (1997). Strange waves in coupled-oscillator arrays: mapping approach. Phys. Rev. Lett. 78, 1683–1686

[B16] Danzl P. Hespanha J. Moehlis J. (2009). Event-based minimum-time control of oscillatory neuron models. Biol. Cybern. 101, 387–399 10.1007/s00422-009-0344-3 19911192

[B17] Deniau J.-M. Degos B. Bosch C. Maurice N. (2010). Deep brain stimulation mechanisms: beyond the concept of local functional inhibition. Eur. J. Neurosci. 32, 1080–1091 10.1111/j.1460-9568.2010.07413.x 21039947

[B18] Ermentrout B. (1996). Type i membranes, phase resetting curves, and synchrony. Neural Comput. 8, 979–1001 8697231 10.1162/neco.1996.8.5.979

[B19] Ermentrout B. Kopell N. (1991). Multiple pulse interactions and averaging in systems of coupled neural oscillators. J. Math. Biol. 29, 195–217

[B20] Ernst U. Pawelzik K. Geisel T. (1995). Synchronization induced by temporal delays in pulse-coupled oscillators. Phys. Rev. Lett. 74, 1570–1573 10.1103/PhysRevLett.74.1570 10059062

[B21] Farries M. A. Wilson C. J. (2012a). Biophysical basis of the phase response curve of subthalamic neurons with generalization to other cell types. J. Neurophysiol. 108, 1838–1855 10.1152/jn.00054.2012 22786959 PMC3774581

[B22] Farries M. A. Wilson C. J. (2012b). Phase response curves of subthalamic neurons measured with synaptic input and current injection. J. Neurophysiol. 108, 1822–1837 10.1152/jn.00053.2012 22786957 PMC3545003

[B23] Feldman D. E. (2000). Timing-based LTP and LTD at vertical inputs to layer II/III pyramidal cells in rat barrel cortex. Neuron 27, 45–56 10.1016/S0896-6273(00)00008-8 10939330

[B24] Feng X. J. Greenwald B. Rabitz H. Shea-Brown E. Kosut R. (2007a). Toward closed-loop optimization of deep brain stimulation for parkinson's disease: concepts and lessons from a computational model. J. Neural Eng. 4, L14–L21 10.1088/1741-2560/4/2/L03 17409470

[B24a] Feng X. J. Shea-Brown E. Greenwald B. Kosut R. Rabitz H. (2007b). Optimal deep brain stimulation of the subthalamic nucleus – a computational study. J. Comput. Neurosci. 23, 265–282 10.1007/s10827-007-0031-0 17484043

[B26] Freund H.-J. (2005). Long-term effects of deep brain stimulation in Parkinson's disease. Brain 128, 2222–2223 10.1093/brain/awh634 16183664

[B27] Gerstner W. Kempter R. van Hemmen J. L. Wagner H. (1996). A neuronal learning rule for sub-millisecond temporal coding. Nature 383, 76–78 10.1038/383076a0 8779718

[B28] Gillies A. Willshaw D. (2004). Models of the subthalamic nucleus: the importance of intranuclear connectivity. Med. Eng. Phys. 26, 723–732 This issue contains a special section on Neuromodelling. 10.1016/j.medengphy.2004.06.003 15564109

[B29] Gillies A. J. Willshaw D. J. (1998). A massively connected subthalamic nucleus leads to the generation of widespread pulses. Proc. R. Soc. B 265, 2101–2109 10.1098/rspb.1998.0546 9842737 PMC1689499

[B30] Goel P. Ermentrout B. (2002). Synchrony, stability, and firing patterns in pulse-coupled oscillators. Physica D 163, 191–216

[B31] Gradinaru V. Mogri M. Thompson K. R. Henderson J. M. Deisseroth K. (2009). Optical deconstruction of parkinsonian neural circuitry. Science 324, 354–359 10.1126/science.1167093 19299587 PMC6744370

[B32] Guckenheimer J. (1975). Isochrons and phaseless sets. J. Math. Biol. 1, 259–273 10.1007/BF01273747 28303309

[B33] Hansel D. Mato G. Meunier C. (1993). Phase dynamics of weakly coupled Hodgkin-Huxley neurons. Europhys. Lett. 23, 367–372

[B34] Hauptmann C. Popovych O. Tass P. A. (2005). Effectively desynchronizing deep brain stimulation based on a coordinated delayed feedback stimulation via several sites: a computational study. Biol. Cybern. 93, 463–470 10.1007/s00422-005-0020-1 16240125

[B35] Hauptmann C. Tass P. A. (2007). Therapeutic rewiring by means of desynchronizing brain stimulation. Biosystems 89, 173–181 10.1016/j.biosystems.2006.04.015 17184901

[B36] Hoppensteadt F. Izhikevich E. (1997). Weakly Connected Neural Networks. New York, NY: Springer-Verlag

[B37] Iwahori N. (1978). Golgi study on subthalamic nucleus of cat. J. Compar. Neurol. 182, 383–397 10.1002/cne.901820303 721967

[B38] Kane A. Hutchison W. D. Hodaie M. Lozano A. M. Dostrovsky J. O. (2009). Enhanced synchronization of thalamic theta band local field potentials in patients with essential tremor. Exp. Neurol. 217, 171–176 10.1016/j.expneurol.2009.02.005 19233174

[B39] Kang G. Lowery M. M. (2011). Effect of subthalamic nucleus interconnectivity at deep brain stimulation onset and offset: a simulation study, in Proceedings of the 33rd Annual International Conference of the IEEE Engineering-in-Medicine-and-Biology-Society (EMBS) (Boston: IEEE), 7107–7110 10.1109/IEMBS.2011.6091796 22255976

[B40] Kita H. Chang H. T. Kitai S. T. (1983). The morphology of intracellularly labeled rat subthalamic neurons – a light microscopic analysis. J. Compar. Neurol. 215, 245–257 10.1002/cne.902150302 6304154

[B41] Krachkovskyi V. Popovych O. V. Tass P. A. (2006). Stimulus-locked responses of two phase oscillators coupled with delayed feedback. Phys. Rev. E 73:066220 10.1103/PhysRevE.73.066220 16906959

[B42] Kumar R. Lozano A. M. Sime E. Lang A. E. (2003). Long-term follow-up of thalamic deep brain stimulation for essential and parkinsonian tremor. Neurology 61, 1601–1604 14663050 10.1212/01.wnl.0000096012.07360.1c

[B43] Kuramoto Y. (1984). Chemical Oscillations, Waves, and Turbulence. Berlin: Springer

[B44] Kuramoto Y. (1997). Phase- and center-manifold reductions for large populations of coupled oscillators with application to non-locally coupled systems. Int. J. Bif. Chaos 7, 789–805

[B45] Lagarias J. Reeds J. A. Wright M. H. Wright P. E. (1998). Convergence properties of the Nelder-Mead simplex method in low dimensions. SIAM J. Optimiz. 9, 112–147

[B46] Lenz F. A. Kwan H. C. Martin R. L. Tasker R. R. Dostrovsky J. O. Lenz Y. E. (1994). Single-unit analysis of the human ventral thalamic nuclear group – tremor-related activity in functionally identified cells. Brain 117, 531–543 10.1093/brain/117.3.531 8032863

[B47] Levesque J. C. Parent A. (2005). Gabaergic interneurons in human subthalamic nucleus. Mov. Disord. 20, 574–584 10.1002/mds.20374 15645534

[B48] Levy R. Hutchison W. D. Lozano A. M. Dostrovsky J. O. (2000). High-frequency synchronization of neuronal activity in the subthalamic nucleus of parkinsonian patients with limb tremor. J. Neurosci. 20, 7766–7775 11027240 10.1523/JNEUROSCI.20-20-07766.2000 PMC6772896

[B49] Limousin P. Speelman J. D. Gielen F. Janssens M. (1999). Multicentre European study of thalamic stimulation in parkinsonian and essential tremor. J. Neurol. Neurosurg. Psychiatry 66, 289–296 10.1136/jnnp.66.3.289 10084526 PMC1736277

[B50] Lücken L. Yanchuk S. (2012). Two-cluster bifurcations in systems of globally pulse-coupled oscillators. Physica D 241, 350–359

[B51] Lysyansky B. Popovych O. V. Tass P. A. (2011a). Desynchronizing anti-resonance effect of m:n on-off coordinated reset stimulation. J. Neural Eng. 8:036019 10.1088/1741-2560/8/3/036019 21555848

[B51b] Lysyansky B. Popovych O. V. Tass P. A. (2011b). Multi-frequency activation of neuronal networks by coordinated reset stimulation. Interface Focus 1, 75–85 10.1098/rsfs.2010.0010 22419975 PMC3262242

[B53] Makeig S. Westerfield M. Jung T. P. Enghoff S. Townsend J. Courchesne E. (2002). Dynamic brain sources of visual evoked responses. Science 295, 690–694 10.1126/science.1066168 11809976

[B54] Markram H. Lóbke J. Frotscher M. Sakmann B. (1997). Regulation of synaptic efficacy by coincidence of postsynaptic APs and EPSPs. Science 275, 213–215 10.1126/science.275.5297.213 8985014

[B55] Mormann F. Lehnertz K. David P. Elger C. E. (2000). Mean phase coherence as a measure for phase synchronization and its application to the eeg of epilepsy patients. Physica D 144, 358–369 12636720

[B56] Nabi A. Moehlis J. (2011). Single input optimal control for globally coupled neuron networks. J. Neural Eng. 8:065008 10.1088/1741-2560/8/6/065008 22056380

[B57] Neiman A. Russell D. Yakusheva T. DiLullo A. Tass P. A. (2007). Response clustering in transient stochastic synchronization and desynchronization of coupled neuronal bursters. Phys. Rev. E 76:021908 10.1103/PhysRevE.76.021908 17930066

[B58] Nini A. Feingold A. Slovin H. Bergmann H. (1995). Neurons in the globus pallidus do not show correlated activity in the normal monkey, but phase-locked oscillations appear in the MPTP model of parkinsonism. J. Neurophysiol. 74, 1800–1805 8989416 10.1152/jn.1995.74.4.1800

[B59] Okuda K. (1993). Variety and generality of clustering in globally coupled oscillators. Physica D 63, 424–436

[B60] Oprisan S. Prinz A. Canavier C. (2004). Phase resetting and phase locking in hybrid circuits of one model and one biological neuron. Biophys. J. 87, 2283 10.1529/biophysj.104.046193 15454430 PMC1304653

[B61] Paydarfar D. Eldridge F. L. (1987). Phase resetting and dysrhythmic responses of the respiratory oscillator. Am. J. Physiol. 252, R55–R62 3812730 10.1152/ajpregu.1987.252.1.R55

[B62] Pikovsky A. Rosenblum M. Kurths J. (2001). Synchronization. A Universal Concept in Nonlinear Sciences. Cambridge: Cambridge University Press

[B63] Popovych O. V. Hauptmann C. Tass P. A. (2005). Effective desynchronization by nonlinear delayed feedback. Phys. Rev. Lett. 94:164102 10.1103/PhysRevLett.94.164102 15904229

[B64] Popovych O. V. Hauptmann C. Tass P. A. (2006). Control of neuronal synchrony by nonlinear delayed feedback. Biol. Cybern. 95, 69–85 10.1007/s00422-006-0066-8 16614837

[B65] Popovych O. V. Tass P. A. (2010). Synchronization control of interacting oscillatory ensembles by mixed nonlinear delayed feedback. Phys. Rev. E 82:026204 10.1103/PhysRevE.82.026204 20866890

[B66] Popovych O. V. Tass P. A. (2011). Macroscopic entrainment of periodically forced oscillatory ensembles. Prog. Biophys. Molec. Biol. 105, 98–108 10.1016/j.pbiomolbio.2010.09.018 20875831

[B67] Popovych O. V. Tass P. A. (2012). Desynchronizing electrical and sensory coordinated reset neuromodulation. Front. Hum. Neurosci. 6:58 10.3389/fnhum.2012.00058 22454622 PMC3308339

[B68] Pyragas K. Popovych O. V. Tass P. A. (2007). Controlling synchrony in oscillatory networks with a separate stimulation-registration setup. Europhys. Lett. 80:40002 10.1209/0295-5075/80/40002

[B69] Roberts L. E. Eggermont J. J. Caspary D. M. Shore S. E. Melcher J. R. Kaltenbach J. A. (2010). Ringing ears: the neuroscience of tinnitus. J. Neurosci. 30, 14972–14979 10.1523/JNEUROSCI.4028-10.2010 21068300 PMC3073522

[B70] Rodriguez-Oroz M. C. Obeso J. A. Lang A. E. Houeto J. L. Pollak P. Rehncrona S. (2005). Bilateral deep brain stimulation in parkinson's disease: a multicentre study with 4 years follow-up. Brain 128, 2240–2249 10.1093/brain/awh571 15975946

[B71] Rosenblum M. Pikovsky A. (2004). Controlling synchronization in an ensemble of globally coupled oscillators. Phys. Rev. Lett. 92, 114102 10.1103/PhysRevLett.92.114102 15089140

[B72] Rosin B. Slovik M. Mitelman R. Rivlin-Etzion M. Haber S. N. Israel Z. (2011). Closed-loop deep brain stimulation is superior in ameliorating parkinsonism. Neuron 72, 370–384 10.1016/j.neuron.2011.08.023 22017994

[B73] Sato Y. Okumura K. Ichiki A. Shiino M. (2011). Thermal effects on phase response curves and synchronization transition, in Advances in Neural Networks – ISNN 2011. Lecture Notes in Computer Science, Vol. 6675, eds Liu D. Zhang H. Polycarpou M. Alippi C. He H. (Berlin; Heidelberg: Springer), 287–296

[B74] Schnitzler A. Munks C. Butz M. Timmermann L. Gross J. (2009). Synchronized brain network associated with essential tremor as revealed by magnetoencephalography. Mov. Disord. 24, 1629–1635 10.1002/mds.22633 19514010

[B75] Schultheiss N. W. Edgerton J. R. Jaeger D. (2010). Phase response curve analysis of a full morphological globus pallidus neuron model reveals distinct perisomatic and dendritic modes of synaptic integration. J. Neurosci. 30, 2767–2782 10.1523/JNEUROSCI.3959-09.2010 20164360 PMC2833015

[B76] Shen K. Z. Johnson S. W. (2006). Subthalamic stimulation evokes complex epscs in the rat substantia nigra pars reticulata *in vitro*. J. Physiol. 573, 697–709 10.1113/jphysiol.2006.110031 16613871 PMC1779757

[B77] Silchenko A. N. Adamchic I. Hauptmann C. Tass P. A. (2013). Impact of acoustic coordinated reset neuromodulation on effective connectivity in a neural network of phantom sound. Neuroimage 77, 133–147 10.1016/j.neuroimage.2013.03.013 23528923

[B78] Stiefel K. M. Gutkin B. S. Sejnowski T. J. (2009). The effects of cholinergic neuromodulation on neuronal phase-response curves of modeled cortical neurons. J. Comput. Neurosci. 26, 289–301 10.1007/s10827-008-0111-9 18784991 PMC2857973

[B79] Strogatz S. H. (2000). From kuramoto to crawford: exploring the onset of synchronization in populations of coupled oscillators. Physica D 143, 1–20

[B80] Swift J. W. Strogatz S. H. Wiesenfeld K. (1992). Averaging of globally coupled oscillators. Physica D 55, 239–250

[B81] Tass P. (1999). Phase Resetting in Medicine and Biology. Stochastic Modelling and Data Analysis. Springer Series in Synergetics. Berlin: Springer

[B82] Tass P. (2003a). A model of desynchronizing deep brain stimulation with a demand-controlled coordinated reset of neural subpopulations. Biol. Cybern. 89, 81–88 10.1007/s00422-003-0425-7 12905037

[B82a] Tass P. A. (2003b). Desynchronization by means of a coordinated reset of neural subpopulations – a novel technique for demand-controlled deep brain stimulation. Prog. Theor. Phys. Suppl. 150, 281–296 10.1007/s00422-003-0425-7 12905037

[B84] Tass P. Adamchic I., Freund H.-J. von Stackelberg T. Hauptmann C. (2012a). Counteracting tinnitus by acoustic coordinated reset neuromodulation. Rest. Neurol. Neurosci. 30, 367–374 10.3233/RNN-2012-110218 22414611

[B84a] Tass P. A. Qin L. Hauptmann C. Doveros S. Bezard E. Boraud T. (2012b). Coordinated reset neuromodulation has sustained effects in parkinsonian non-human primates. Ann. Neurol. 72, 816–820 10.1002/ana.23663 23280797

[B86] Tass P. A. (2001a). Effective desynchronization by means of double-pulse phase resetting. Europhys. Lett. 53, 15–21

[B86b] Tass P. A. (2001b). Desynchronizing double-pulse phase resetting and application to deep brain stimulation. Biol. Cybern. 85, 343–354 11721989 10.1007/s004220100268

[B88] Tass P. A. (2002a). Effective desynchronization with a stimulation technique based on soft phase resetting. Europhys. Lett. 57, 164–170

[B89] Tass P. A. (2002b). Desynchronization of brain rhythms with soft phase-resetting techniques. Biol. Cybern. 87, 102–115 10.1007/s00422-002-0322-5 12181586

[B90] Tass P. A. Majtanik M. (2006). Long-term anti-kindling effects of desynchronizing brain stimulation: a theoretical study. Biol. Cybern. 94, 58–66 10.1007/s00422-005-0028-6 16284784

[B84b] Tass P. A. Popovych O. V. (2012). Unlearning tinnitus-related cerebral synchrony with acoustic coordinated reset stimulation: theoretical concept and modelling. Biol. Cybern. 106, 27–36 10.1007/s00422-012-0479-5 22350536

[B92] Tass P. A. Silchenko A. N. Hauptmann C. Barnikol U. B. Speckmann E. J. (2009). Long-lasting desynchronization in rat hippocampal slice induced by coordinated reset stimulation. Phys. Rev. E 80:011902 10.1103/PhysRevE.80.011902 19658724

[B93] Thorne J. D. De Vos M. Viola F. C. Debener S. (2011). Cross-modal phase reset predicts auditory task performance in humans. J. Neurosci. 31, 3853–3861 10.1523/JNEUROSCI.6176-10.2011 21389240 PMC6622791

[B94] Volkmann J. (2004). Deep brain stimulation for the treatment of parkinson's disease. J. Clin. Neurophysiol. 21, 6–17 15097290 10.1097/00004691-200401000-00003

[B95] van Vreeswijk C. Abbott L. F. Ermentrout G. B. (1994). When inhibition not excitation synchronizes neural firing. J. Comput. Neurosci. 1, 313–321 8792237 10.1007/BF00961879

[B96] Weisz N. Moratti S. Meinzer M. Dohrmann K. Elbert T. (2005). Tinnitus perception and distress is related to abnormal spontaneous brain activity as measured by magnetoencephalography. PLoS Med. 2:e153 10.1371/journal.pmed.0020153 15971936 PMC1160568

[B97] Weisz N. Muller S. Schlee W. Dohrmann K. Hartmann T. Elbert T. (2007). The neural code of auditory phantom perception. J. Neurosci. 27, 1479–1484 10.1523/JNEUROSCI.3711-06.2007 17287523 PMC6673575

[B98] Wilson C. J. Beverlin B. Netoff T. I. (2011). Chaotic desynchronization as the therapeutic mechanism of deep brain stimulation. Front. Syst. Neurosci. 5:50 10.3389/fnsys.2011.00050 21734868 PMC3122072

[B99] Wilson C. L. Puntis M. Lacey M. G. (2004). Overwhelmingly asynchronous firing of rat subthalamic nucleus neurones in brain slices provides little evidence for intrinsic interconnectivity. Neuroscience 123, 187–200 10.1016/j.neuroscience.2003.09.008 14667453

[B100] Winfree A. (2001). The Geometry of Biological Time. New York, NY: Springer

[B101] Winfree A. T. (1977). Phase-control of neural pacemakers. Science 197, 761–763 10.1126/science.887919 887919

[B102] Wittenberg G. M. Wang S. S.-H. (2006). Malleability of spike-timing-dependent plasticity at the CA3-CA1 synapse. J. Neurosci. 26, 6610–6617 10.1523/JNEUROSCI.5388-05.2006 16775149 PMC6674029

[B103] Zillmer R. Livi R. Politi A. Torcini A. (2007). Stability of the splay state in pulse-coupled networks. Phys. Rev. E 76:046102 10.1103/PhysRevE.76.046102 17995055

